# Supplementary material for: Economic evaluation of the cost of different methods of retesting chlamydia positive individuals in England
Source: BMJ Open. 2019 Mar 23;9(3):e024828. doi: 10.1136/bmjopen-2018-024828 (PMC6475158; doi:10.1136/bmjopen-2018-024828)
Supplement: Supplementary file 1 [file bmjopen-2018-024828supp001.pdf]

Appendix

An economic evaluation of the cost of different methods of retesting chlamydia positive individuals in England

Looker, K. J., Buitendam, E., Woodhall, S. C., Hollis, E., Ong, K.-J., Saunders, J., Dunbar, K. and Turner, K. M. E.

Appendix Table 1 Chlamydia retest costs by recall method

Activity	Price per unit (item/ minute of staff time)	Recall method											
		1. Client-led		2. Reminder card		3. SMS invitation		4. Phone invitation		5. Automatic postal test kit		6. Advice at follow-up & SMS	
		Units	Cost	Units	Cost	Units	Cost	Units	Cost	Units	Cost	Units	Cost
**PATIENT OFFERED RETEST**													
Nurse-led conversation about retesting at first diagnosis and issue subsequent reminders													
Nurse band 5/6/7/8[2]	£ 0.89	3	£ 2.68	3	£ 2.68	3	£ 2.68	13	£ 11.60	3	£ 2.68	13	£ 11.60
Blend admin/clerical[1]	£ 0.53	0	£ -	0	£ -	5	£ 2.64	5	£ 2.64	0	£ -	10	£ 5.27
Reminder card	£ 0.10	0	£ -	1	£ 0.10	0	£ -	0	£ -	0	£ -	0	£ -
SMS text message	£ 0.10	0	£ -	0	£ -	1	£ 0.10	0	£ -	0	£ -	1	£ 0.10
Phone call[2]	£ 0.07	0	£ -	0	£ -	0	£ -	3	£ 0.21	0	£ -	3	£ 0.21
**PATIENT DELIVERED RETEST[8]**													
Register, meet and greet													
Blend admin/clerical[3]	£ 0.53	3.8	£ 2.00	3.8	£ 2.00	3.8	£ 2.00	3.8	£ 2.00	0.0	£ -	3.8	£ 2.00
Actual retest													
Consultation - blend of Community SRH staff (N2 to Doctor)[3]	£ 1.06	6.8	£ 7.22	6.8	£ 7.22	6.8	£ 7.22	6.8	£ 7.22	0.0	£ -	6.8	£ 7.22
Blend admin/clerical[1,4]	£ 0.53	1.2	£ 0.63	1.2	£ 0.63	1.2	£ 0.63	1.2	£ 0.63	5	£ 2.64	1.2	£ 0.63
Health professional-led retest - blend of Community SRH staff (N2 to Doctor)[3]	£ 1.06	4.56	£ 4.81	4.56	£ 4.81	4.56	£ 4.81	4.56	£ 4.81	0	£ -	4.56	£ 4.81
Gloves[3]	£ 0.05	0.76	£ 0.03	0.76	£ 0.03	0.76	£ 0.03	0.76	£ 0.03	0	£ -	0.76	£ 0.03
Lab request form with bag	£ 0.10	1	£ 0.10	1	£ 0.10	1	£ 0.10	1	£ 0.10	1	£ 0.10	1	£ 0.10

Sample collection instructions	£ 0.05	1	£ 0.05	1	£ 0.05	1	£ 0.05	1	£ 0.05	1	£ 0.05	1	£ 0.05
Transport tube	£ 0.26	1	£ 0.26	1	£ 0.26	1	£ 0.26	1	£ 0.26	1	£ 0.26	1	£ 0.26
Urine pot, sterile collection	£ 0.23	0.7	£ 0.16	0.7	£ 0.16	0.7	£ 0.16	0.7	£ 0.16	0.7	£ 0.16	0.7	£ 0.16
Urine specimen container (PCR tube and pipette)	£ 1.04	0.7	£ 0.73	0.7	£ 0.73	0.7	£ 0.73	0.7	£ 0.73	0.7	£ 0.73	0.7	£ 0.73
Vulvo-vaginal swab	£ 0.16	0.3	£ 0.05	0.3	£ 0.05	0.3	£ 0.05	0.3	£ 0.05	0.3	£ 0.05	0.3	£ 0.05
Postage/packaging[4]	£ 0.89	0.24	£ 0.21	0.24	£ 0.21	0.24	£ 0.21	0.24	£ 0.21	1	£ 0.89	0.24	£ 0.21
Return envelope and postage[4]	£ 0.89	0.24	£ 0.21	0.24	£ 0.21	0.24	£ 0.21	0.24	£ 0.21	1	£ 0.89	0.24	£ 0.21
Health promotion/Q&A[6]													
Health professional-led discussion - blend of Community SRH staff (N2 to Doctor)	£ 1.06	6	£ 6.33	6	£ 6.33	6	£ 6.33	6	£ 6.33	6	£ 6.33	6	£ 6.33
KY lubricant	£ 0.30	2	£ 0.60	2	£ 0.60	2	£ 0.60	2	£ 0.60	2	£ 0.60	2	£ 0.60
STI literature	£ 0.06	3	£ 0.18	3	£ 0.18	3	£ 0.18	3	£ 0.18	3	£ 0.18	3	£ 0.18
Male condom	£ 0.06	10	£ 0.58	10	£ 0.58	10	£ 0.58	10	£ 0.58	10	£ 0.58	10	£ 0.58
**RETEST PROCESSED AND RESULTS GIVEN**													
Pathology													
Lab processing	£ 12.51	1	£ 12.51	1	£ 12.51	1	£ 12.51	1	£ 12.51	1	£ 12.51	1	£ 12.51
Results management (retest negatives)													
Nurse band 5/6	£ 0.75	6	£ 4.50	6	£ 4.50	6	£ 4.50	6	£ 4.50	6	£ 4.50	6	£ 4.50
Letter notification	£ 0.58	0.02	£ 0.01	0.02	£ 0.01	0.02	£ 0.01	0.02	£ 0.01	0.02	£ 0.01	0.02	£ 0.01
Phone call	£ 0.07	0.03	£ 0.00	0.03	£ 0.00	0.03	£ 0.00	0.03	£ 0.00	0.03	£ 0.00	0.03	£ 0.00
SMS text message	£ 0.10	0.95	£ 0.10	0.95	£ 0.10	0.95	£ 0.10	0.95	£ 0.10	0.95	£ 0.10	0.95	£ 0.10
Results management (retest positives/equivocal)													
Nurse band 5/6/7/8	£ 0.89	15	£ 13.38	15	£ 13.38	15	£ 13.38	15	£ 13.38	15	£ 13.38	15	£ 13.38
Letter notification	£ 0.58	0.05	£ 0.03	0.05	£ 0.03	0.05	£ 0.03	0.05	£ 0.03	0.05	£ 0.03	0.05	£ 0.03
Phone call	£ 0.07	0.05	£ 0.00	0.05	£ 0.00	0.05	£ 0.00	0.05	£ 0.00	0.05	£ 0.00	0.05	£ 0.00
SMS text message	£ 0.10	0.9	£ 0.09	0.9	£ 0.09	0.9	£ 0.09	0.9	£ 0.09	0.9	£ 0.09	0.9	£ 0.09
Treatment[7]	£ 1.80	1	£ 1.80	1	£ 1.80	1	£ 1.80	1	£ 1.80	1	£ 1.80	1	£ 1.80
Follow-up call[5]													
Blend admin/clerical[1,4]	£ 0.53	5	£ 2.64	5	£ 2.64	5	£ 2.64	5	£ 2.64	5	£ 2.64	5	£ 2.64
Nurse band 5/6[2]	£ 0.75	10	£ 7.50	10	£ 7.50	10	£ 7.50	10	£ 7.50	10	£ 7.50	10	£ 7.50
Phone call[2]	£ 0.07	3	£ 0.21	3	£ 0.21	3	£ 0.21	3	£ 0.21	3	£ 0.21	3	£ 0.21

Shaded entries were taken directly from the basic cost of a (first) chlamydia test[1] which is reproduced here under a Creative Commons licence:

© Pathway Analytics

All other costs are either amended costs from Pathway Analytics (see below for explanation), or costs added in.

[1]Any contact at a distance with client further to the initial retest conversation is assumed to require 5 minutes of admin time to retrieve and update the client's details on a database. [2]A phone call is assumed to be 3 minutes in length but requiring 10 minutes of nurse time to accommodate chasing time. [3]Applicable to clinic retesting: these costs are removed entirely where retesting involves postal testing only, and reduced

proportionally for the remaining recall methods to allow for some clients opting for postal retesting. [4]Applies to postal kits: includes postage to client's address and return postage[2], and associated admin time for sending out a kit. [5]Positives only. [6]It is assumed that all clients will received these at some point including those opting for retesting by post. [7]4 x 250mg of azithromycin in tablet form[3]. VAT, dispensing costs and costs associated with a test for cure are not included. [8]Number of units for clinic vs postal testing kit costs obtained by multiplying base costs by the percentage using each.

Appendix Figure 1 Retesting pathway

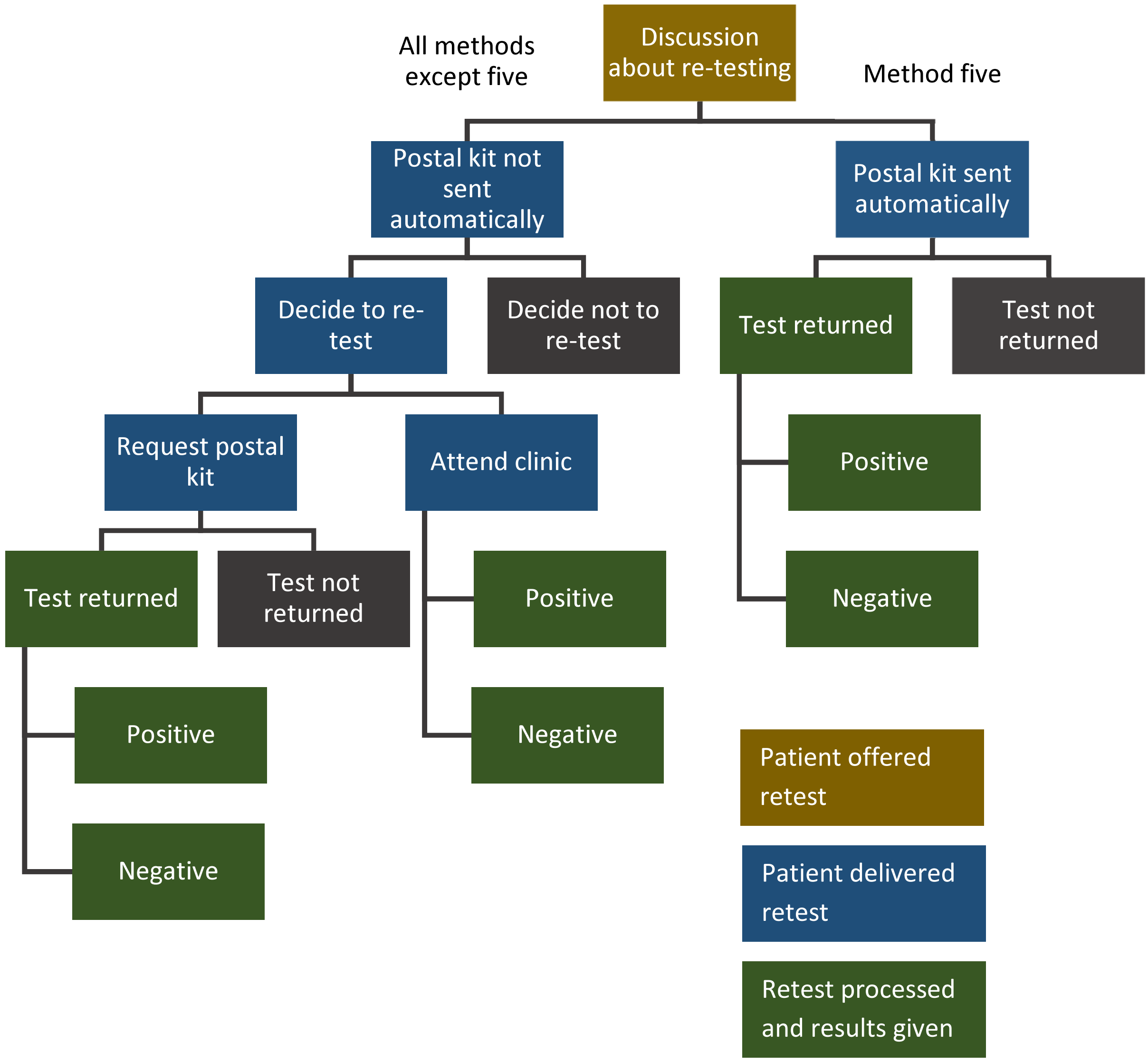

Appendix Table 2 Parameter values

Parameter	Baseline value (10-14 weeks since treatment for first infection)		Sensitivity value (10-26 weeks since treatment for first infection)		Reference
Retest uptake (i.e., percentage of (first) positives who choose to accept retesting) (all methods except method five)[1]	1. Client-led	5%	1. Client-led	15%	NCSP audit report[4] and NCSP audit data provided by PHE
	2. Reminder card	4%	2. Reminder card	19%	
	3. SMS invitation	9%	3. SMS invitation	21%	
	4. Phone invitation	7%	4. Phone invitation	17%	
	6. Advice at follow-up & SMS	13%	6. Advice at follow-up & SMS	25%	
Percentage of those retesting who choose to attend a clinic for a retest (all methods except method five)[2]	76%		73%		NCSP audit data provided by PHE
Percentage of those retesting who choose to request a postal kit (all methods except method five)	=100-76% =24%		=100-73% =27%		
Postal test kit return rate (requested kits)	67%		67%		Retesting pilot[5]
Postal test kit return rate (kits sent out automatically; method five)	5. Automatic postal test kit	10%	5. Automatic postal test kit	23%	NCSP audit report[4] and NCSP audit data provided by PHE
Chlamydia retest positivity[3]	12%		16%		NCSP audit report[4] and NCSP audit data provided by PHE

[1]Obtained by fitting to overall retest rates from the audit (i.e., accounting for non-return of requested postal kits); [2]The audit only has data on percentage of *completed* retests obtained from clinic testing vs postal testing (for those instances where a kit was not sent out automatically), not percentage of those who opt for a retest at a clinic among *all* retesters (i.e., including all those who request a kit, some of whom do not return the kit); [3]Average over the six most commonly-used methods.

References

1. Pathway Analytics: Pathway Prices for Integrated Sexual Health Tariffs *118. T3 Chlamydia, gonorrhoea and syphilis tests https://www.pathwayanalytics.com/pathways/26-t3-chlamydia-gonorrhoea-syphilis-tests/118-t3-chlamydia-gonorrhoea-and-syphilis-tests Accessed 2013*.
2. *Royal Mail: Get a price http://www.royalmail.com/price-finder Accessed 14/08/2015*.
3. *British National Formulary: Azithromycin http://www.evidence.nhs.uk/formulary/bnf/current/5-infections/51-antibacterial-drugs/515-macrolides/azithromycin Accessed 02/02/2016*.
4. *Re-testing of those who tested positive for chlamydia: National audit report https://www.gov.uk/government/uploads/system/uploads/attachment_data/file/471585/NCSPre-testingauditfinalversion.pdf Accessed 13/09/2016*.
5. Angel, G., et al., *An observational study to evaluate three pilot programmes of retesting chlamydia-positive individuals within 6 months in the South West of England.* BMJ Open, 2016. **5**(10): p. e007455.
