# Supplementary material for: Economic evaluation of the cost of different methods of retesting chlamydia positive individuals in England
Source: BMJ Open. 2019 Mar 23;9(3):e024828. doi: 10.1136/bmjopen-2018-024828 (PMC6475158; doi:10.1136/bmjopen-2018-024828)
Supplement: Author's manuscript [file bmjopen-2018-024828.draft_revisions.pdf]

BMJ Open is committed to open peer review. As part of this commitment we make the peer review history of every article we publish publicly available.

When an article is published we post the peer reviewers' comments and the authors' responses online. We also post the versions of the paper that were used during peer review. These are the versions that the peer review comments apply to.

The versions of the paper that follow are the versions that were submitted during the peer review process. They are not the versions of record or the final published versions. They should not be cited or distributed as the published version of this manuscript.

BMJ Open is an open access journal and the full, final, typeset and author-corrected version of record of the manuscript is available on our site with no access controls, subscription charges or pay-per-view fees (http://bmjopen.bmj.com).

If you have any questions on BMJ Open's open peer review process please email info.bmjopen@bmj.com

BMJ Open

A comparison of the cost of different methods of retesting chlamydia positive individuals in England

Journal:	*BMJ Open*
Manuscript ID	bmjopen-2018-024828
Article Type:	Research
Date Submitted by the Author:	15-Jun-2018
Complete List of Authors:	Looker, Katharine; University of Bristol, Population Health Sciences, Bristol Medical School<br>Buitendam, Erna; Public Health England, HIV & STI Department, National Infection Service<br>Woodhall, Sarah; Public Health England, HIV & STI; UCL, Research Department of Infection and Population Health<br>Hollis, Emma; Public Health England, HIV/STI<br>Ong, Koh Jun; Public Health England, National Infections Service - HIV/STI<br>Saunders, John; Public Health England, HIV & STI Department; University College London, Research Department of Infection and Population Health<br>Dunbar, Kevin; Public Health England, HIV & STI Department<br>Turner, Katy; Bristol University, School of Social and Community Medicine
Keywords:	Public health < INFECTIOUS DISEASES, HEALTH SERVICES ADMINISTRATION & MANAGEMENT, HEALTH ECONOMICS

SCHOLARONE™
Manuscripts

A comparison of the cost of different methods of retesting chlamydia positive individuals in England

Looker, K. J.[1]*, Buitendam, E.[2], Woodhall, S. C.[2], Hollis, E.[2], Ong, K.-J.[2], Saunders, J.[2], Dunbar, K.[2] and Turner, K. M. E.[1]

[1]Population Health Sciences, Bristol Medical School, University of Bristol, Bristol, United Kingdom

[2]HIV & STI Department, National Infection Service, Public Health England, United Kingdom

*Corresponding author. Correspondence should be sent to:

Dr Katharine Looker

Population Health Sciences, Bristol Medical School

University of Bristol

Oakfield House, Oakfield Grove

Bristol BS8 2BN

United Kingdom

katharine.looker@bristol.ac.uk

For peer review only

For peer review only - http://bmjopen.bmj.com/site/about/guidelines.xhtml

ABSTRACT

Objectives

The National Chlamydia Screening Programme (NCSP) in England opportunistically screens eligible individuals for chlamydia infection. In 2013, the NCSP recommended retesting three months after treatment following a positive test result. However, no guidance was given on how local areas should recall individuals for retesting. Here we compare cost estimates for different recall methods to inform the optimal delivery of retesting programmes.

Methods

We estimated the cost of chlamydia retesting for each of the six most commonly-used recall methods in 2014 based on existing cost estimates of a chlamydia screen. Proportions accepting retesting, opting for retesting by post, returning postal testing kits and retesting positive were informed by 2014 NCSP audit data. Costs were "sense-checked" by health professionals.

Results

We estimated the cost of the chlamydia retest pathway, including treatment and follow-up call, to be between £45-£70 per completed test. At the lower end this compared favourably to the cost of a clinic-based screen. After adjusting for incomplete uptake, and non-return of postal kits, the cost estimate rose to £109-£289 per test offered. The most economical method in terms of the adjusted cost per retest was no active recall, as gains in retest rates with active recall did not outweigh the higher cost. Nurse-led client contact by phone was particularly uneconomical, as was sending out postal testing kits automatically. However, if the cost of sending an SMS could be lowered by reducing administration costs, the higher retest rate achieved by an SMS invitation could make this the most economical way of delivering retesting.

Conclusions

Retesting using the least-intensive methods (no active recall/recall by single SMS) is more economical than more intensive methods such as recalling by phone and automatically sending out postal kits. However, patient choice and local accessibility of services should be taken into consideration in planning.

Strengths and limitations of this study

- We compared the cost of the chlamydia retest pathway in England across the five most commonly-used methods of recalling individuals for retesting, to enable local service planners to assess whether they are delivering retesting economically or should consider an alternative approach.
- Our cost estimates included both clinic retesting, and retesting using postal kits.
- We incorporated incomplete uptake, and non-return of postal kits, to estimate cost based on actual patterns of use.
- We did not specifically look at the cost of on-line testing, nor account for the effect of demographic and clinical factors on retest uptake by recall method.

For peer review only - http://bmjopen.bmj.com/site/about/guidelines.xhtml

- Extra detail on the methods is available in the Appendix.

For peer review only

For peer review only - http://bmjopen.bmj.com/site/about/guidelines.xhtml

INTRODUCTION

Chlamydia trachomatis (chlamydia) is the most commonly-diagnosed bacterial sexually-transmitted infection (STI) in England[1]. Since 2003 there has been a National Chlamydia Screening Programme (NCSP) in England which opportunistically tests sexually-active 15-24 year olds[2]. NCSP guidelines recommend retesting three months after treatment for chlamydia[3]. British Association for Sexual Health and HIV (BASHH) national guidelines recommend retesting under 25 year olds three to six months after treatment[4]. No guidance is given by either the NCSP or BASHH on how local areas should recall individuals for retesting, which can be done in many ways. The 2014 NCSP retesting audit[5] found that the most common methods of recalling individuals for retesting were as follows: (1) conversation with client at time of test result with no further reminder (32%); (2) reminder card given to client at time of test result with no further reminder (1%); (3) client sent text message when retest due (29%); (4) client invited by phone call when retest due (8%); testing kit posted to client's chosen address when retest due (5%); and (6) retesting advised at follow up call with client - text message sent at 3 months (19%).

Previous estimates exist for the cost of a clinic-based chlamydia screen[6, 7]. However, to our knowledge there are no estimates of the cost of a chlamydia retest, and how this varies by recall method. Specifically, we do not know the best way to balance getting the optimal number of people to retest versus the additional cost of delivering invitations or reminders to retest. Understanding how the cost of retesting varies depending on the approach taken is critical for optimal programme delivery. Here we present cost estimates for different recall methods, firstly for the retest pathway itself, and then for the adjusted cost per retest, allowing for incomplete uptake, and non-return of postal kits, to impact on cost.

METHODS

We estimated the cost of chlamydia retesting using Microsoft Excel 2016 for each of the six most commonly-used recall methods reported in the 2014 NCSP retesting audit[5] (Table 1) as follows. First, we entered existing cost estimates for a chlamydia test from Pathway Analytics (costed for clinic-based chlamydia screening for 2011), which excluding a follow-up call was around £45[6] (Appendix Table 1). We used this costing as given. We then added additional costs to reflect costs specifically associated with retesting, such as a nurse-led conversation about retesting after diagnosis, and issuing retest invitations/reminders (e.g., by phone or text message [SMS]). In addition, we amended the clinic-based chlamydia test costs to allow for postal testing.

For each of the six recall methods, we costed both the retest pathway, and the adjusted cost per retest (Appendix Figure 1). The adjusted cost per retest accounts for incomplete uptake, and non-return of postal kits. For all methods except method five (automatic postal testing kit) we allowed clients to choose either to attend a clinic for retesting, or to request a postal testing kit. Thus, for methods one to four, and method six, we incorporated the following parameters: retest uptake, the proportion who opt for postal testing, and the return rate of requested kits. The 2014 NCSP audit[5] measured overall retest rates, which were calculated from the number that attended a clinic for a retest or returned a postal testing kit, divided by the total number recalled for retesting. This is different to retest uptake, which is the number that attended a clinic for a retest or ordered or were sent a postal testing kit, divided by the total number recalled for retesting. Retest uptake for each of

For peer review only - http://bmjopen.bmj.com/site/about/guidelines.xhtml

the six recall methods was fitted to overall retest rates from the 2014 NCSP audit[5], taking a value of 24% for the proportion of clients who opt for postal testing (also from the audit), and a value of 67% for the return rate of requested kits[8]. For method five, uptake was equivalent to overall retest rate and was simply the return rate of postal kits (10%) from the 2014 NCSP audit[5]. Chlamydia retest positivity (12%) was taken from the NCSP audit[5], and was averaged over all six recall methods due to small numbers by individual method. For a table of parameter values see Appendix Table 2. We also calculated the cost and adjusted cost per retest positive, i.e., the cost of finding one positive retest incorporating the cost of other, negative retests.

The time frame for calculating the parameter values was 10-14 weeks, corresponding to NCSP guidance for retesting. We sense-checked our retesting costs (Appendix Table 1) with health professionals. We conducted two sensitivity analyses. In the first sensitivity analysis we replaced the parameters for the retesting pathway with those obtained from data for retesting done between 10-26 weeks (corresponding to BASHH guidance) (Appendix Table 2). This simply allows more time for clients to retest: there is no additional contact with clients to remind them to retest. In the second sensitivity analysis we altered staff salary costs from nurse bands to administrator bands for nurse-based costs associated with phone invitations to retest, managing a retest negative, and a follow-up call at three months for those retesting positive (leaving the nurse-based costs associated with the initial retest conversation and managing a retest positive unchanged). The purpose of this was to show the difference in price that could be achieved if administrative staff instead of nurses contacted clients by phone, except where a lower band of advisor might not be appropriate.

During the study, we also had access to unpublished data from the 2017 NCSP retesting audit (Erna Buitendam, personal communication). For the six most commonly-used recall methods in the 2014 audit, we found that retest rates significantly increased for method one (client-led) and method five (automatic postal test kit) between the 2014 and 2017 audits (p>0.05). Therefore, it was not appropriate to combine the data for 2014 and 2017, and we restricted our analyses to 2014 data only. However, we carried out an analysis of whether retest positivity was statistically-significantly different for no active recall (method one) versus active recall (methods three and six) using both 2014 and 2017 audit data, since there was no statistically-significant difference in the positivity rates for each of these methods when comparing 2014 and 2017 data.

Table 1 Chlamydia retest costs by recall method

	Recall method					
	**1. Client-led**	**2. Reminder card**	**3. SMS invitation**	**4. Phone invitation**	**5. Automatic postal test kit**	**6. Advice at follow-up & SMS**
Number of retest invitations by each method (%), N=2853[1] (NCSP audit, 2014[5])	**912 (32%)**	**27 (1%)**	**840 (29%)**	**227 (8%)**	**130 (5%)**	**528 (19%)**
Description	Conversation with client at time of test result with no further reminder	Reminder card given to client at time of test result with no further reminder	Client sent text message when retest due	Client invited by phone call when retest due	Testing kit posted to client's chosen address when retest due	Retesting advised at follow up call with client - text message sent at 3 months

**COSTS USING BASELINE PARAMETERS (10-14 weeks since treatment for first infection)**						
**Cost of chlamydia retesting pathway[2]**						
Cost of offering retesting	**£2.68**	**£2.78**	**£5.42**	**£14.44**	**£2.68**	**£17.18**
Cost of delivering retest	**£24.16**	**£24.16**	**£24.16**	**£24.16**	**£13.45**	**£24.16**
Cost of processing retest and giving results	**£28.71**	**£28.71**	**£28.71**	**£28.71**	**£28.71**	**£28.71**
**TOTAL COST**	**£55.54**	**£55.64**	**£58.28**	**£67.31**	**£44.83**	**£70.05**
**Cost per retest positive**	**£481**	**£482**	**£505**	**£583**	**£389**	**£607**
**Retest rate**	**5%**	**4%**	**8%**	**6%**	**10%**	**12%**
**Adjusted cost per retest incorporating incomplete uptake/non-return of kits**	**£109**	**£130**	**£120**	**£289**	**£190**	**£195**
**Adjusted cost per retest positive incorporating incomplete uptake/non-return of kits**	**£946**	**£1,126**	**£1,039**	**£2,506**	**£1,646**	**£1,686**
**COSTS USING LONGER TIME WINDOW FOR RETESTING (10-26 weeks since treatment for first infection)**						
**Total cost of chlamydia retesting pathway**	**£55.38**	**£55.48**	**£58.12**	**£67.15**	**£45.32**	**£69.89**
**Cost per retest positive**	**£344**	**£345**	**£361**	**£417**	**£282**	**£435**
**Retest rate**	**15%**	**19%**	**21%**	**17%**	**23%**	**25%**
**Adjusted cost per retest incorporating incomplete uptake/non-return of kits**	**£73**	**£71**	**£82**	**£142**	**£99**	**£126**
**Adjusted cost per retest positive incorporating incomplete uptake/non-return of kits**	**£456**	**£440**	**£508**	**£883**	**£616**	**£780**
**COSTS IF ADMINISTRATORS USED INSTEAD OF NURSES**						
**Total cost of chlamydia retesting pathway**	**£52.13**	**£52.23**	**£54.87**	**£60.24**	**£41.42**	**£62.98**
**Cost per retest positive**	**£452**	**£453**	**£476**	**£522**	**£359**	**£546**
**Adjusted cost per retest incorporating incomplete uptake/non-return of kits**	**£106**	**£126**	**£117**	**£227**	**£187**	**£161**
**Adjusted cost per retest positive incorporating incomplete uptake/non-return of kits**	**£917**	**£1,096**	**£1,010**	**£1,963**	**£1,617**	**£1,399**

[1]Other methods or method not recorded account for the remaining 7% (N=189) of retests. [2]Some costs were taken (and some subsequently amended) from the basic cost of a (first) chlamydia test[6] which is under a Creative Commons licence: © Pathway Analytics

RESULTS

The estimated cost of the chlamydia retest pathway ranged from £45-£70 per completed test, while the cost per retest positive ranged from £389-£607 (Table 1). Posting testing kits automatically with no further reminder (method five) was the cheapest recall method, while methods involving inviting clients by phone to retest (methods four and six) were the most expensive. After adjusting for incomplete uptake and non-return of postal kits, the cost per chlamydia retest ranged from £109-£289 per test offered, while the cost per retest positive ranged from £946-£2,506. Here, the most economical recall method in terms of the adjusted cost per retest was no active recall (method one). The most expensive methods were still those involving inviting clients by phone to retest (methods four and six). This was despite these methods achieving higher retest rates (6% and 12% for methods four and six, respectively) compared to no active recall (5%). Sending postal testing kits out automatically (method five) was also an uneconomical way of delivering a retest, due to the cost of

For peer review only

non-returned kits. An SMS invitation (method three) increased retest rates for comparatively small cost but was only the most economical if administration time for sending an SMS was reduced from five to three minutes (results not shown). Retest positivity was not statistically-significantly different for no active recall (method one) versus active recall (methods three and six) when 2014 and 2017 audit data were combined.

Extending the retesting period to 10-26 weeks did not impact substantially on the chlamydia retesting pathway cost (range £45-£70) (Table 1). However, the adjusted cost per retest incorporating incomplete uptake and non-return of kits was substantially lower (range £71-£126), as was the adjusted cost per retest positive (range £440-£883), than with a tighter retest window, particularly for automatically sending out postal kits (method five). However, this assumed positivity was higher for the 10-26 week window across all methods. In any case, methods with no or else minimal active recall were still the most economical. Replacing nurse bands with administrator bands only had a substantial impact on costs for those methods where clients were contacted by phone to recall for retest (Table 1).

DISCUSSION

The estimated cost of the chlamydia retest pathway ranged from £45-£70 per completed test, which at the cheapest end was very similar to the cost of a clinic-based chlamydia screen[6, 7]. Important differences were seen when uptake and kit return rates were varied. This is because successfully completed retests effectively absorbed the cost of incomplete retests. Here, the most economical recall method involved no active recall after the initial retest conversation. Sending out postal testing kits automatically was an expensive way of doing retesting because of wastage of kits. However, the most expensive methods involved contacting clients by phone to invite them to retest, primarily because of nurse time required.

When the retesting window was increased from 10-14 weeks to 10-26 weeks, all methods of recall had a reduced adjusted cost per retest, due in part to higher positivity for 10-26 weeks. However, a longer time window means there is further potential for onward transmission, so it is important clients are counselled on the best time to retest.

Active recall increased retest rates but this did not outweigh the additional cost. We assumed that sending an SMS involved administration time to retrieve clients' details from a database. If the associated cost was reduced, then an SMS invitation or reminder could be an economical way of increasing retest rates. We did not find any evidence that retest positivity was different for active recall versus no active recall, meaning there is no evidence that active recall merely results in more negatives being tested. However, evidence from a retesting pilot in South-West England did show that those who retested without being actively recalled had higher chlamydia retest positivity than those who were actively recalled[8]. Furthermore, the unpublished 2017 audit data showed a statistically-significant increase in the recall rate for client-led retesting for 10-14 weeks compared to 2014 (Erna Buitendam, personal communication), which could make no active recall even more economical than shown here.

Our analysis was done for the pathway cost of testing for chlamydia alone[6]. Where chlamydia testing is done at the same time as testing for other STIs (such as gonorrhoea), the proportionate cost of testing for chlamydia will be reduced. Another consideration is that since our analysis was

For peer review only - http://bmjopen.bmj.com/site/about/guidelines.xhtml

carried out, the estimated pathway cost has fallen, which will reduce costs further across all methods of retesting. Lastly, online testing with automated recall is likely to be the most economical method of all, but was beyond the scope of this analysis. A further limitation is we did not account for the effect on retest uptake of different demographic and clinical factors, such as gender, location of services, country of birth, sexual orientation and presence of symptoms.

Our analysis suggests that no active recall after the initial retest conversation is the most economical way of retesting, although an SMS invitation or reminder could be considered. Patient choice and accessibility of services should still be taken into consideration for local delivery planning, and it is important that retest uptake is monitored as this determines how economical retesting is.

CONTRIBUTORS

KJL undertook the itemisation and costing, analysed the results and drafted the manuscript. KMET oversaw the study and provided advice as needed. EB and SW provided audit data and advised on parameterisation. K-JO helped with sources for costs. KJL, EB, SW, EH, K-JO, JS, KD and KMET all contributed to the progress of the study and manuscript revisions.

CONFLICT OF INTEREST

The authors declare there are no conflicts of interest.

ACKNOWLEDGEMENTS

KJL and KMET thank the National Institute for Health Research (NIHR) Health Protection Research Unit (HPRU) in Evaluation of Interventions at the University of Bristol, in partnership with Public Health England, for research support. We would like to thank Rose Tobin (North East and North Central London Adult Critical Care Network, Royal Free London NHS Foundation Trust), Stephanie Rumsey (East Cheshire NHS Trust), Jan Cremer (Essex Partnership University NHS Foundation Trust) and Stephanie Sawyer (London Borough of Bromley Public Health) for sense-checking the costs, and Pathway Analytics for data on the cost of a chlamydia test.

FUNDING

This work was supported by the NIHR Health Protection Research Unit in Evaluation of Interventions at the University of Bristol, in partnership with Public Health England. This funder had no role in the writing of the manuscript nor the decision to submit it for publication. The views expressed are those of the authors and not necessarily those of the NHS, the NIHR, the Department of Health or Public Health England.

REFERENCES

1. *National chlamydia screening programme (NCSP): data tables* *https://www.gov.uk/government/statistics/national-chlamydia-screening-programme-ncsp-data-tables Accessed 13/09/2016*.
2. *NCSP: programme overview https://www.gov.uk/government/publications/ncsp-programme-overview Accessed 13/09/2016*.

For peer review only - http://bmjopen.bmj.com/site/about/guidelines.xhtml

3. *Consultation Report: Routine offer of re-test to young adults testing positive for chlamydia (Consultation findings and evidence summary)* https://www.gov.uk/government/uploads/system/uploads/attachment_data/file/508364/NCSP_Report_Consultation_on_Re-testing_of_Positive_Chlamydia_Cases_August_2013_FINAL.pdf *Accessed 14/09/2016*.
4. Nwokolo, N.C., et al. *2015 UK national guideline for the management of infection with Chlamydia trachomatis* http://www.bashh.org/documents/UK%20Chlamydia%20Guidelines%202015.pdf *Accessed 14/09/2016*.
5. *Re-testing of those who tested positive for chlamydia: National audit report* https://www.gov.uk/government/uploads/system/uploads/attachment_data/file/471585/NCSPre-testingauditfinalversion.pdf *Accessed 13/09/2016*.
6. Pathway Analytics: Pathway Prices for Integrated Sexual Health Tariffs *118. T3 Chlamydia, gonorrhoea and syphilis tests* https://www.pathwayanalytics.com/pathways/26-t3-chlamydia-gonorrhoea-syphilis-tests/118-t3-chlamydia-gonorrhoea-and-syphilis-tests *Accessed 2013*.
7. Turner, K., et al., *Costs and cost effectiveness of different strategies for chlamydia screening and partner notification: an economic and mathematical modelling study.* BMJ, 2011. **342**: p. c7250.
8. Angel, G., et al., *An observational study to evaluate three pilot programmes of retesting chlamydia-positive individuals within 6 months in the South West of England.* BMJ Open, 2016. **5**(10): p. e007455.

For peer review only - http://bmjopen.bmj.com/site/about/guidelines.xhtml

Appendix

A comparison of the cost of different methods of retesting chlamydia positive individuals in England

Looker, K. J., Buitendam, E., Woodhall, S. C., Hollis, E., Ong, K.-J., Saunders, J., Dunbar, K. and Turner, K. M. E.

Appendix Table 1 Chlamydia retest costs by recall method

Activity	Price per unit (item/ minute of staff time)	Recall method											
		1. Client-led		2. Reminder card		3. SMS invitation		4. Phone invitation		5. Automatic postal test kit		6. Advice at follow-up & SMS	
		Units	Cost	Units	Cost	Units	Cost	Units	Cost	Units	Cost	Units	Cost
**PATIENT OFFERED RETEST**													
Nurse-led conversation about retesting at first diagnosis and issue subsequent reminders													
Nurse band 5/6/7/8[2]	£ 0.89	3	£ 2.68	3	£ 2.68	3	£ 2.68	13	£ 11.60	3	£ 2.68	13	£ 11.60
Blend admin/clerical[1]	£ 0.53	0	£ -	0	£ -	5	£ 2.64	5	£ 2.64	0	£ -	10	£ 5.27
Reminder card	£ 0.10	0	£ -	1	£ 0.10	0	£ -	0	£ -	0	£ -	0	£ -
SMS text message	£ 0.10	0	£ -	0	£ -	1	£ 0.10	0	£ -	0	£ -	1	£ 0.10
Phone call[2]	£ 0.07	0	£ -	0	£ -	0	£ -	3	£ 0.21	0	£ -	3	£ 0.21
**PATIENT DELIVERED RETEST[8]**													
Register, meet and greet													
Blend admin/clerical[3]	£ 0.53	3.8	£ 2.00	3.8	£ 2.00	3.8	£ 2.00	3.8	£ 2.00	0.0	£ -	3.8	£ 2.00
Actual retest													
Consultation - blend of Community SRH staff (N2 to Doctor)[3]	£ 1.06	6.8	£ 7.22	6.8	£ 7.22	6.8	£ 7.22	6.8	£ 7.22	0.0	£ -	6.8	£ 7.22
Blend admin/clerical[1,4]	£ 0.53	1.2	£ 0.63	1.2	£ 0.63	1.2	£ 0.63	1.2	£ 0.63	5	£ 2.64	1.2	£ 0.63
Health professional-led retest - blend of Community SRH staff (N2 to Doctor)[3]	£ 1.06	4.56	£ 4.81	4.56	£ 4.81	4.56	£ 4.81	4.56	£ 4.81	0	£ -	4.56	£ 4.81
Gloves[3]	£ 0.05	0.76	£ 0.03	0.76	£ 0.03	0.76	£ 0.03	0.76	£ 0.03	0	£ -	0.76	£ 0.03
Lab request form with bag	£ 0.10	1	£ 0.10	1	£ 0.10	1	£ 0.10	1	£ 0.10	1	£ 0.10	1	£ 0.10

For peer review only - http://bmjopen.bmj.com/site/about/guidelines.xhtml

Sample collection instructions	£ 0.05	1	£ 0.05	1	£ 0.05	1	£ 0.05	1	£ 0.05	1	£ 0.05	1	£ 0.05
Transport tube	£ 0.26	1	£ 0.26	1	£ 0.26	1	£ 0.26	1	£ 0.26	1	£ 0.26	1	£ 0.26
Urine pot, sterile collection	£ 0.23	0.7	£ 0.16	0.7	£ 0.16	0.7	£ 0.16	0.7	£ 0.16	0.7	£ 0.16	0.7	£ 0.16
Urine specimen container (PCR tube and pipette)	£ 1.04	0.7	£ 0.73	0.7	£ 0.73	0.7	£ 0.73	0.7	£ 0.73	0.7	£ 0.73	0.7	£ 0.73
Vulvo-vaginal swab	£ 0.16	0.3	£ 0.05	0.3	£ 0.05	0.3	£ 0.05	0.3	£ 0.05	0.3	£ 0.05	0.3	£ 0.05
Postage/packaging[4]	£ 0.89	0.24	£ 0.21	0.24	£ 0.21	0.24	£ 0.21	0.24	£ 0.21	1	£ 0.89	0.24	£ 0.21
Return envelope and postage[4]	£ 0.89	0.24	£ 0.21	0.24	£ 0.21	0.24	£ 0.21	0.24	£ 0.21	1	£ 0.89	0.24	£ 0.21
Health promotion/Q&A[6]													
Health professional-led discussion - blend of Community SRH staff (N2 to Doctor)	£ 1.06	6	£ 6.33	6	£ 6.33	6	£ 6.33	6	£ 6.33	6	£ 6.33	6	£ 6.33
KY lubricant	£ 0.30	2	£ 0.60	2	£ 0.60	2	£ 0.60	2	£ 0.60	2	£ 0.60	2	£ 0.60
STI literature	£ 0.06	3	£ 0.18	3	£ 0.18	3	£ 0.18	3	£ 0.18	3	£ 0.18	3	£ 0.18
Male condom	£ 0.06	10	£ 0.58	10	£ 0.58	10	£ 0.58	10	£ 0.58	10	£ 0.58	10	£ 0.58
**RETEST PROCESSED AND RESULTS GIVEN**													
Pathology													
Lab processing	£ 12.51	1	£ 12.51	1	£ 12.51	1	£ 12.51	1	£ 12.51	1	£ 12.51	1	£ 12.51
Results management (retest negatives)													
Nurse band 5/6	£ 0.75	6	£ 4.50	6	£ 4.50	6	£ 4.50	6	£ 4.50	6	£ 4.50	6	£ 4.50
Letter notification	£ 0.58	0.02	£ 0.01	0.02	£ 0.01	0.02	£ 0.01	0.02	£ 0.01	0.02	£ 0.01	0.02	£ 0.01
Phone call	£ 0.07	0.03	£ 0.00	0.03	£ 0.00	0.03	£ 0.00	0.03	£ 0.00	0.03	£ 0.00	0.03	£ 0.00
SMS text message	£ 0.10	0.95	£ 0.10	0.95	£ 0.10	0.95	£ 0.10	0.95	£ 0.10	0.95	£ 0.10	0.95	£ 0.10
Results management (retest positives/equivocal)													
Nurse band 5/6/7/8	£ 0.89	15	£ 13.38	15	£ 13.38	15	£ 13.38	15	£ 13.38	15	£ 13.38	15	£ 13.38
Letter notification	£ 0.58	0.05	£ 0.03	0.05	£ 0.03	0.05	£ 0.03	0.05	£ 0.03	0.05	£ 0.03	0.05	£ 0.03
Phone call	£ 0.07	0.05	£ 0.00	0.05	£ 0.00	0.05	£ 0.00	0.05	£ 0.00	0.05	£ 0.00	0.05	£ 0.00
SMS text message	£ 0.10	0.9	£ 0.09	0.9	£ 0.09	0.9	£ 0.09	0.9	£ 0.09	0.9	£ 0.09	0.9	£ 0.09
Treatment[7]	£ 1.80	1	£ 1.80	1	£ 1.80	1	£ 1.80	1	£ 1.80	1	£ 1.80	1	£ 1.80
Follow-up call[5]													
Blend admin/clerical[1,4]	£ 0.53	5	£ 2.64	5	£ 2.64	5	£ 2.64	5	£ 2.64	5	£ 2.64	5	£ 2.64
Nurse band 5/6[2]	£ 0.75	10	£ 7.50	10	£ 7.50	10	£ 7.50	10	£ 7.50	10	£ 7.50	10	£ 7.50
Phone call[2]	£ 0.07	3	£ 0.21	3	£ 0.21	3	£ 0.21	3	£ 0.21	3	£ 0.21	3	£ 0.21

Shaded entries were taken directly from the basic cost of a (first) chlamydia test[1] which is reproduced here under a Creative Commons licence:

© Pathway Analytics

All other costs are either amended costs from Pathway Analytics (see below for explanation), or costs added in.

[1]Any contact at a distance with client further to the initial retest conversation is assumed to require 5 minutes of admin time to retrieve and update the client's details on a database. [2]A phone call is assumed to be 3 minutes in length but requiring 10 minutes of nurse time to accommodate chasing time. [3]Applicable to clinic retesting: these costs are removed entirely where retesting involves postal testing only, and reduced

proportionally for the remaining recall methods to allow for some clients opting for postal retesting. [4]Applies to postal kits: includes postage to client's address and return postage[2], and associated admin time for sending out a kit. [5]Positives only. [6]It is assumed that all clients will received these at some point including those opting for retesting by post. [7]4 x 250mg of azithromycin in tablet form[3]. VAT, dispensing costs and costs associated with a test for cure are not included. [8]Number of units for clinic vs postal testing kit costs obtained by multiplying base costs by the percentage using each.

For peer review only

Appendix Figure 1 Retesting pathway

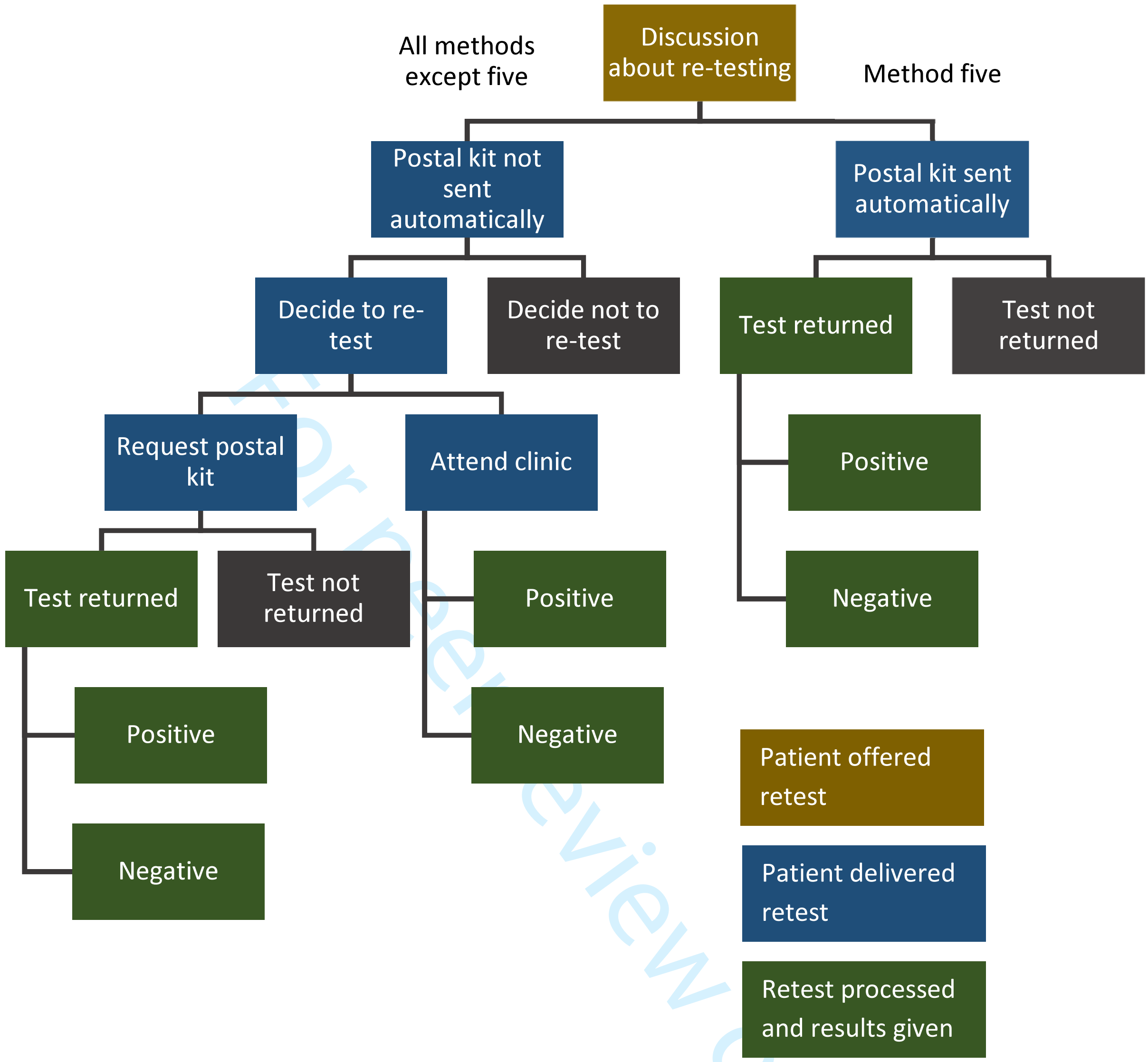

Appendix Table 2 Parameter values

Parameter	Baseline value (10-14 weeks since treatment for first infection)		Sensitivity value (10-26 weeks since treatment for first infection)		Reference
Retest uptake (i.e., percentage of (first) positives who choose to accept retesting) (all methods except method five)[1]	1. Client-led	5%	1. Client-led	15%	NCSP audit report[4] and NCSP audit data provided by PHE
	2. Reminder card	4%	2. Reminder card	19%	
	3. SMS invitation	9%	3. SMS invitation	21%	
	4. Phone invitation	7%	4. Phone invitation	17%	
	6. Advice at follow-up & SMS	13%	6. Advice at follow-up & SMS	25%	
Percentage of those retesting who choose to attend a clinic for a retest (all methods except method five)[2]	76%		73%		NCSP audit data provided by PHE
Percentage of those retesting who choose to request a postal kit (all methods except method five)	=100-76% =24%		=100-73% =27%		
Postal test kit return rate (requested kits)	67%		67%		Retesting pilot[5]
Postal test kit return rate (kits sent out automatically; method five)	5. Automatic postal test kit	10%	5. Automatic postal test kit	23%	NCSP audit report[4] and NCSP audit data provided by PHE
Chlamydia retest positivity[3]	12%		16%		NCSP audit report[4] and NCSP audit data provided by PHE

[1]Obtained by fitting to overall retest rates from the audit (i.e., accounting for non-return of requested postal kits); [2]The audit only has data on percentage of *completed* retests obtained from clinic testing vs postal testing (for those instances where a kit was not sent out automatically), not percentage of those who opt for a retest at a clinic among *all* retesters (i.e., including all those who request a kit, some of whom do not return the kit); [3]Average over the six most commonly-used methods.

References

1. Pathway Analytics: Pathway Prices for Integrated Sexual Health Tariffs *118. T3 Chlamydia, gonorrhoea and syphilis tests* https://www.pathwayanalytics.com/pathways/26-t3-chlamydia-gonorrhoea-syphilis-tests/118-t3-chlamydia-gonorrhoea-and-syphilis-tests *Accessed 2013*.
2. *Royal Mail: Get a price* http://www.royalmail.com/price-finder *Accessed 14/08/2015*.
3. *British National Formulary: Azithromycin* http://www.evidence.nhs.uk/formulary/bnf/current/5-infections/51-antibacterial-drugs/515-macrolides/azithromycin *Accessed 02/02/2016*.
4. *Re-testing of those who tested positive for chlamydia: National audit report* https://www.gov.uk/government/uploads/system/uploads/attachment_data/file/471585/NCSPre-testingauditfinalversion.pdf *Accessed 13/09/2016*.
5. Angel, G., et al., *An observational study to evaluate three pilot programmes of retesting chlamydia-positive individuals within 6 months in the South West of England.* BMJ Open, 2016. **5**(10): p. e007455.

For peer review only

BMJ Open

An economic evaluation of the cost of different methods of retesting chlamydia positive individuals in England

Journal:	*BMJ Open*
Manuscript ID	bmjopen-2018-024828.R1
Article Type:	Research
Date Submitted by the Author:	11-Oct-2018
Complete List of Authors:	Looker, Katharine; University of Bristol, Population Health Sciences, Bristol Medical School<br>Buitendam, Erna; Public Health England, HIV & STI Department, National Infection Service<br>Woodhall, Sarah; Public Health England, HIV & STI; UCL, Research Department of Infection and Population Health<br>Hollis, Emma; Public Health England, HIV/STI<br>Ong, Koh Jun; Public Health England, National Infections Service - HIV/STI<br>Saunders, John; Public Health England, HIV & STI Department; University College London, Research Department of Infection and Population Health<br>Dunbar, Kevin; Public Health England, HIV & STI Department<br>Turner, Katy; Bristol University, School of Social and Community Medicine
**Primary Subject Heading**:	Sexual health
Secondary Subject Heading:	Health services research, Health economics, Public health, Infectious diseases
Keywords:	Public health < INFECTIOUS DISEASES, HEALTH SERVICES ADMINISTRATION & MANAGEMENT, HEALTH ECONOMICS

SCHOLARONE™
Manuscripts

An economic evaluation of the cost of different methods of retesting chlamydia positive individuals in England

Looker, K. J.[1]*, Buitendam, E.[2], Woodhall, S. C.[2], Hollis, E.[2], Ong, K.-J.[2], Saunders, J.[2], Dunbar, K.[2] and Turner, K. M. E.[1]

[1]Population Health Sciences, Bristol Medical School, University of Bristol, Bristol, United Kingdom

[2]HIV & STI Department, National Infection Service, Public Health England, United Kingdom

*Corresponding author. Correspondence should be sent to:

Dr Katharine Looker

Population Health Sciences, Bristol Medical School

University of Bristol

Oakfield House, Oakfield Grove

Bristol BS8 2BN

United Kingdom

katharine.looker@bristol.ac.uk

For peer review only

For peer review only - http://bmjopen.bmj.com/site/about/guidelines.xhtml

ABSTRACT

Objectives

The National Chlamydia Screening Programme in England opportunistically screens eligible individuals for chlamydia infection. Retesting is recommended three months after treatment following a positive test result, but no guidance is given on how local areas should recall individuals for retesting. Here we compare cost estimates for different recall methods to inform the optimal delivery of retesting programmes.

Design

Economic evaluation

Setting

England

Methods

We estimated the cost of chlamydia retesting for each of the six most commonly-used recall methods in 2014 based on existing cost estimates of a chlamydia screen. Proportions accepting retesting, opting for retesting by post, returning postal testing kits and retesting positive were informed by 2014 NCSP audit data. Health professionals "sense-checked" the costs.

Primary and secondary outcomes

Cost and adjusted cost per chlamydia retest; cost and adjusted cost per chlamydia retest positive.

Results

We estimated the cost of the chlamydia retest pathway, including treatment/follow-up call, to be between £45-£70 per completed test. At the lower end this compared favourably to the cost of a clinic-based screen. Cost per retest positive was £389-£607. After adjusting for incomplete uptake, and non-return of postal kits, the cost rose to £109-£289 per completed test (cost per retest positive: £946-£2,506). The most economical method in terms of adjusted cost per retest was no active recall, as gains in retest rates with active recall did not outweigh the higher cost. Nurse-led client contact by phone was particularly uneconomical, as was sending out postal testing kits automatically.

Conclusions

Retesting without active recall is more economical than more intensive methods such as recalling by phone and automatically sending out postal kits. If sending an SMS could be automated, this could be the most economical way of delivering retesting. However, patient choice and local accessibility of services should be taken into consideration in planning.

For peer review only

For peer review only - http://bmjopen.bmj.com/site/about/guidelines.xhtml

Strengths and limitations of this study

We compared the cost of the chlamydia retest pathway in England across the five most commonly-used methods of recalling individuals for retesting, to enable local service planners to assess whether they are delivering retesting economically or should consider an alternative approach.

Our cost estimates included both clinic retesting, and retesting using postal kits.

We incorporated incomplete uptake, and non-return of postal kits, to estimate cost based on actual patterns of use.

We did not specifically look at the effect of clinical factors on cost, although no active recall is likely to be associated with similar or higher test positivity than active recall.

We also did not consider other important factors besides cost such as the demography of the population: for example, automatically sending out postal kits might be the only feasible option in rural areas, and indeed, on-line testing, which was not considered in our analysis, is likely to be the most economical method of all.

INTRODUCTION

Chlamydia trachomatis (chlamydia) is the most commonly-diagnosed bacterial sexually-transmitted infection (STI) in England[1]. Since 2003 there has been a National Chlamydia Screening Programme (NCSP) in England which opportunistically tests sexually-active 15-24 year olds[2]. NCSP guidelines recommend retesting three months after treatment for chlamydia[3]. British Association for Sexual Health and HIV (BASHH) national guidelines recommend retesting under 25 year olds three to six months after treatment[4]. No guidance is given by either the NCSP or BASHH on how local areas should recall individuals for retesting, which can be done in many ways. The 2014 NCSP retesting audit[5] found that the most common methods of recalling individuals for retesting were as follows: (1) conversation with client at time of test result with no further reminder (32%); (2) reminder card given to client at time of test result with no further reminder (1%); (3) client sent text message when retest due (29%); (4) client invited by phone call when retest due (8%); (5) testing kit posted to client's chosen address when retest due (5%); and (6) retesting advised at follow up call with client - text message sent at 3 months (19%). The audit also measured overall retest rates for each recall method, which were calculated from the number that attended a clinic for a retest or returned a postal testing kit, divided by the total number recalled for retesting. This is different to retest uptake, which is the number that attended a clinic for a retest or *ordered* or were sent a postal testing kit, divided by the total number recalled for retesting, which has cost implications. Retest uptake was not measured by the audit. Unpublished data from the 2017 NCSP retesting audit (Erna Buitendam, personal communication) showed that, for the six most commonly-used recall methods in the 2014 audit, retest rates significantly increased for method one (client-led) and method five (automatic postal test kit) between the 2014 and 2017 audits.

For peer review only - http://bmjopen.bmj.com/site/about/guidelines.xhtml

Previous estimates exist for the cost of a clinic-based chlamydia screen[6, 7]. However, to our knowledge there are no estimates of the cost of a chlamydia retest, and how this varies by recall method. Specifically, we do not know the best way to balance getting the optimal number of people to retest versus the additional cost of delivering invitations or reminders to retest. Understanding how the cost of retesting varies depending on the approach taken is critical for optimal programme delivery. Here we present cost estimates for different recall methods in England, firstly for the retest pathway itself, and then for the adjusted cost per retest, allowing for incomplete uptake, and non-return of postal kits, to impact on cost.

METHODS

We estimated the cost of chlamydia retesting in England using Microsoft Excel 2016 for each of the six most commonly-used recall methods reported in the 2014 NCSP retesting audit[5] (Table 1) as follows. First, we entered existing cost estimates for a chlamydia test from Pathway Analytics (costed for clinic-based chlamydia screening for 2011), which excluding a follow-up call was around £45[6] (Appendix Table 1). We used this costing as given. We then added additional costs to reflect costs specifically associated with retesting, such as a nurse-led conversation about retesting after diagnosis, and issuing retest invitations/reminders (e.g., by phone or text message [SMS]). In addition, we amended the clinic-based chlamydia test costs to allow for postal testing. Costs were then totalled across the following cost categories: cost of offering retesting, cost of delivering retest, and cost of processing retest and giving results, as well as overall.

For each of the six recall methods, we costed both the retest pathway, and the adjusted cost per retest (Appendix Figure 1). The adjusted cost per retest accounts for incomplete uptake, and non-return of postal kits, within each cost category. For all methods except method five (automatic postal testing kit) we allowed clients to choose either to attend a clinic for retesting, or to request a postal testing kit. Thus, for methods one to four, and method six, we incorporated the following parameters: retest uptake, the proportion who opt for postal testing, and the return rate of requested kits. Retest uptake for each of the six recall methods was fitted to overall retest rates from the 2014 NCSP audit[5], taking a value of 24% for the proportion of clients who opt for postal testing (also from the audit), and a value of 67% for the return rate of requested kits[8]. For method five, uptake was equivalent to overall retest rate and was simply the return rate of postal kits (10%) from the 2014 NCSP audit[5]. Chlamydia retest positivity (12%), which informs the relative weight given to the cost of managing a positive retest result versus managing a negative retest result in the average cost of the chlamydia retesting pathway, was taken from the NCSP audit[5], and was averaged over all six recall methods due to small numbers by individual method. We also calculated the cost and adjusted cost per retest positive, i.e., the cost of finding one positive retest incorporating the cost of other, negative retests, by dividing test costs by the chlamydia positivity. For a table of parameter values see Appendix Table 2.

The time frame for calculating the parameter values was 10-14 weeks, corresponding to NCSP guidance for retesting. We sense-checked our retesting costs (Appendix Table 1) with health professionals. We conducted two sensitivity analyses. In the first sensitivity analysis we replaced the parameters for the retesting pathway with those obtained from data for retesting done between 10-26 weeks (corresponding to BASHH guidance) (Appendix Table 2). This simply allows more time for clients to retest: there is no

For peer review only - http://bmjopen.bmj.com/site/about/guidelines.xhtml

additional contact with clients to remind them to retest. In the second sensitivity analysis we altered staff salary costs from nurse bands to administrator bands for nurse-based costs associated with phone invitations to retest, managing a retest negative, and a follow-up call at three months for those retesting positive (leaving the nurse-based costs associated with the initial retest conversation and managing a retest positive unchanged). The purpose of this was to show the difference in price that could be achieved if administrative staff instead of nurses contacted clients by phone, except where a lower band of advisor might not be appropriate.

Since retest rates significantly increased for method one (client-led) and method five (automatic postal test kit) between the 2014 and (unpublished) 2017 audits (p>0.05), we restricted our analyses to 2014 data only. However, we carried out an analysis of whether retest positivity was statistically-significantly different for no active recall (method one) versus active recall (methods three and six) using both 2014 and 2017 audit data, since there was no statistically-significant difference in the positivity rates for each of these methods when comparing 2014 and 2017 data.

Patient and Public Involvement

Patients and the public were not involved in this analysis.

For peer review only - http://bmjopen.bmj.com/site/about/guidelines.xhtml

Table 1 Chlamydia retest costs by recall method

	Recall method					
	1. Client-led	2. Reminder card	3. SMS invitation	4. Phone invitation	5. Automatic postal test kit	6. Advice at follow-up & SMS
Number of retest invitations by each method (%), N=2853[1] (NCSP audit, 2014[5])	**912 (32%)**	**27 (1%)**	**840 (29%)**	**227 (8%)**	**130 (5%)**	**528 (19%)**
Description	Conversation with client at time of test result with no further reminder	Reminder card given to client at time of test result with no further reminder	Client sent text message when retest due	Client invited by phone call when retest due	Testing kit posted to client's chosen address when retest due	Retesting advised at follow up call with client - text message sent at 3 months
**COSTS USING BASELINE PARAMETERS (10-14 weeks since treatment for first infection)**						
**Cost of chlamydia retesting pathway[2]**						
Cost of offering retesting	**£2.68**	**£2.78**	**£5.42**	**£14.44**	**£2.68**	**£17.18**
Cost of delivering retest	**£24.16**	**£24.16**	**£24.16**	**£24.16**	**£13.45**	**£24.16**
Cost of processing retest and giving results	**£28.71**	**£28.71**	**£28.71**	**£28.71**	**£28.71**	**£28.71**
**TOTAL COST**	**£55.54**	**£55.64**	**£58.28**	**£67.31**	**£44.83**	**£70.05**
**Cost per retest positive**	**£481**	**£482**	**£505**	**£583**	**£389**	**£607**
**Retest uptake**	**5%**	**4%**	**9%**	**7%**	**10%**	**13%**
**Retest rate**	**5%**	**4%**	**8%**	**6%**	**10%**	**12%**
**Adjusted cost per retest incorporating incomplete uptake/non-return of kits**	**£109**	**£130**	**£120**	**£289**	**£190**	**£195**
**Adjusted cost per retest positive incorporating incomplete uptake/non-return of kits**	**£946**	**£1,126**	**£1,039**	**£2,506**	**£1,646**	**£1,686**
**COSTS USING LONGER TIME WINDOW FOR RETESTING (10-26 weeks since treatment for first infection)**						
**Total cost of chlamydia retesting pathway**	**£55.38**	**£55.48**	**£58.12**	**£67.15**	**£45.32**	**£69.89**
**Cost per retest positive**	**£344**	**£345**	**£361**	**£417**	**£282**	**£435**
**Retest update**	**16%**	**20%**	**23%**	**18%**	**23%**	**27%**
**Retest rate**	**15%**	**19%**	**21%**	**17%**	**23%**	**25%**
**Adjusted cost per retest incorporating incomplete uptake/non-return of kits**	**£73**	**£71**	**£82**	**£142**	**£99**	**£126**
**Adjusted cost per retest positive incorporating incomplete uptake/non-return of kits**	**£456**	**£440**	**£508**	**£883**	**£616**	**£780**
**COSTS IF ADMINISTRATORS USED INSTEAD OF NURSES**						
**Total cost of chlamydia retesting pathway**	**£52.13**	**£52.23**	**£54.87**	**£60.24**	**£41.42**	**£62.98**
**Cost per retest positive**	**£452**	**£453**	**£476**	**£522**	**£359**	**£546**
**Adjusted cost per retest incorporating incomplete uptake/non-return of kits**	**£106**	**£126**	**£117**	**£227**	**£187**	**£161**

For peer review only - http://bmjopen.bmj.com/site/about/guidelines.xhtml

**Adjusted cost per retest positive incorporating incomplete uptake/non-return of kits**	**£917**	**£1,096**	**£1,010**	**£1,963**	**£1,617**	**£1,399**

[1]Other methods or method not recorded account for the remaining 7% (N=189) of retests. [2]Some costs were taken (and some subsequently amended) from the basic cost of a (first) chlamydia test[6] which is under a Creative Commons licence:

© Pathway Analytics

RESULTS

The estimated cost of the chlamydia retest pathway ranged from £45-£70 per completed test, while the cost per retest positive ranged from £389-£607 (Table 1). Posting testing kits automatically with no further reminder (method five) was the cheapest recall method, while methods involving inviting clients by phone to retest (methods four and six) were the most expensive. After adjusting for incomplete uptake and non-return of postal kits, the cost per chlamydia retest ranged from £109-£289 per completed test, while the cost per retest positive ranged from £946-£2,506. Here, the most economical recall method in terms of the adjusted cost per retest was no active recall (method one). An SMS invitation (method three) increased retest rates for comparatively small additional cost, however. The most expensive methods were still those involving inviting clients by phone to retest (methods four and six). This was despite these methods achieving higher retest rates (6% and 12% for methods four and six, respectively) compared to no active recall (5%). Sending postal testing kits out automatically (method five) was also an uneconomical way of delivering a retest, due to the cost of non-returned kits. Retest positivity was not statistically-significantly different for no active recall (method one) versus active recall (methods three and six) when 2014 and 2017 audit data were combined.

Extending the retesting period to 10-26 weeks did not impact substantially on the chlamydia retesting pathway cost (range £45-£70) (Table 1). However, the adjusted cost per retest incorporating incomplete uptake and non-return of kits was substantially lower (range £71-£126), as was the adjusted cost per retest positive (range £440-£883), than with a tighter retest window, particularly for automatically sending out postal kits (method five). However, this assumed positivity was higher for the 10-26 week window across all methods. In any case, methods with no or else minimal active recall were still the most economical. Replacing nurse bands with administrator bands only had a substantial impact on costs for those methods where clients were contacted by phone to recall for retest (Table 1).

DISCUSSION

The estimated cost of the chlamydia retest pathway ranged from £45-£70 per completed test, which at the cheapest end was very similar to the cost of a clinic-based chlamydia screen[6, 7]. The cost per retest positive, meanwhile, ranged from £389-£607. Important differences were seen when uptake and kit return rates were varied. This is because successfully completed retests effectively absorbed the cost of incomplete retests. Here, the most economical recall method involved no active recall after the initial retest conversation. Sending out postal testing kits automatically was an expensive way of doing retesting because of wastage of kits. However, the most expensive methods involved contacting clients by phone to invite them to retest, primarily because of nurse time required.

For peer review only - http://bmjopen.bmj.com/site/about/guidelines.xhtml

When the retesting window was increased from 10-14 weeks to 10-26 weeks, all methods of recall had a reduced adjusted cost per retest, due in part to higher positivity for 10-26 weeks. However, a longer time window means there is further potential for onward transmission, so it is important clients are counselled on the best time to retest.

Active recall increased retest rates but this did not outweigh the additional cost. We assumed that sending an SMS involved administration time to retrieve clients' details from a database. In our analysis we considered only the effect of altering staff salary costs from nurse bands to administrator bands for some nurse-based activities. If the time needed to send an SMS could be shortened by automating this process, then an SMS invitation or reminder could be an economical way of increasing retest rates. Conversations with health professionals during the course of our study suggested that a shorter administration time to send an SMS was theoretically feasible. We did not find any evidence that retest positivity was different for active recall versus no active recall, meaning there is no evidence that active recall merely results in more negatives being tested. However, evidence from a retesting pilot in South-West England did show that those who retested without being actively recalled had higher chlamydia retest positivity than those who were actively recalled[8]. Furthermore, the unpublished 2017 audit data showed a statistically-significant increase in the recall rate for client-led retesting for 10-14 weeks compared to 2014 (Erna Buitendam, personal communication), which could make no active recall even more economical than shown here.

Our analysis was done for the pathway cost of testing for chlamydia alone[6]. Where chlamydia testing is done at the same time as testing for other STIs (such as gonorrhoea), the proportionate cost of testing for chlamydia will be reduced. Another consideration is that since our analysis was carried out, the estimated pathway cost has fallen, which will reduce costs further across all methods of retesting. However, cost is not the only important factor to consider. For example, the cheapest recall methods also had the lowest retest rates, although as noted above, active recall may not necessarily identify more infected people if those opting to retest self-select on the basis of their perceived risk or presence of symptoms. We also did not account for the effect on retest uptake of factors such as gender, location of services, country of birth, and sexual orientation. The composition of the population is an important consideration in local planning: a large rural population, for example, might affect how retesting needs to be delivered. Given the much higher return rate for requested postal testing kits compared to kits sent out automatically, online testing with automated recall is likely to be the most economical method of all, but was beyond the scope of this analysis.

Our analysis suggests that no active recall after the initial retest conversation is the most economical way of retesting, although an SMS invitation or reminder could be considered. Patient choice and accessibility of services should still be taken into consideration for local delivery planning and it is important that retest uptake is monitored as this determines how economical retesting is.

CONTRIBUTORS

KJL undertook the itemisation and costing, analysed the results and drafted the manuscript. KMET oversaw the study and provided advice as needed. EB and SW provided audit data and advised on

For peer review only - http://bmjopen.bmj.com/site/about/guidelines.xhtml

parameterisation. K-JO helped with sources for costs. KJL, EB, SW, EH, K-JO, JS, KD and KMET all contributed to the progress of the study and manuscript revisions.

CONFLICT OF INTEREST

The authors declare there are no conflicts of interest.

ACKNOWLEDGEMENTS

KJL and KMET thank the National Institute for Health Research (NIHR) Health Protection Research Unit (HPRU) in Evaluation of Interventions at the University of Bristol, in partnership with Public Health England, for research support. We would like to thank Rose Tobin (North East and North Central London Adult Critical Care Network, Royal Free London NHS Foundation Trust), Stephanie Rumsey (East Cheshire NHS Trust), Jan Cremer (Essex Partnership University NHS Foundation Trust) and Stephanie Sawyer (London Borough of Bromley Public Health) for sense-checking the costs, and Pathway Analytics for data on the cost of a chlamydia test.

FUNDING

This work was supported by the NIHR Health Protection Research Unit in Evaluation of Interventions at the University of Bristol, in partnership with Public Health England. This funder had no role in the writing of the manuscript nor the decision to submit it for publication. The views expressed are those of the authors and not necessarily those of the NHS, the NIHR, the Department of Health or Public Health England.

REFERENCES

1. *National chlamydia screening programme (NCSP): data tables* *https://www.gov.uk/government/statistics/national-chlamydia-screening-programme-ncsp-data-tables Accessed 13/09/2016*.
2. *NCSP: programme overview https://www.gov.uk/government/publications/ncsp-programme-overview Accessed 13/09/2016*.
3. *Consultation Report: Routine offer of re-test to young adults testing positive for chlamydia (Consultation findings and evidence summary) https://www.gov.uk/government/uploads/system/uploads/attachment_data/file/508364/NCSP_Report_Consultation_on_Re-testing_of_Positive_Chlamydia_Cases_August_2013_FINAL.pdf Accessed 14/09/2016*.
4. Nwokolo, N.C., et al. *2015 UK national guideline for the management of infection with Chlamydia trachomatis http://www.bashh.org/documents/UK%20Chlamydia%20Guidelines%202015.pdf Accessed 14/09/2016*.
5. *Re-testing of those who tested positive for chlamydia: National audit report https://www.gov.uk/government/uploads/system/uploads/attachment_data/file/471585/NCSP re-testingauditfinalversion.pdf Accessed 13/09/2016*.
6. Pathway Analytics: Pathway Prices for Integrated Sexual Health Tariffs *118. T3 Chlamydia, gonorrhoea and syphilis tests https://www.pathwayanalytics.com/pathways/26-t3-chlamydia-gonorrhoea-syphilis-tests/118-t3-chlamydia-gonorrhoea-and-syphilis-tests Accessed 2013*.

For peer review only - http://bmjopen.bmj.com/site/about/guidelines.xhtml

7. Turner, K., et al., *Costs and cost effectiveness of different strategies for chlamydia screening and partner notification: an economic and mathematical modelling study.* BMJ, 2011. **342**: p. c7250.

8. Angel, G., et al., *An observational study to evaluate three pilot programmes of retesting chlamydia-positive individuals within 6 months in the South West of England.* BMJ Open, 2016. **5**(10): p. e007455.

For peer review only

For peer review only - http://bmjopen.bmj.com/site/about/guidelines.xhtml

Appendix

An economic evaluation of the cost of different methods of retesting chlamydia positive individuals in England

Looker, K. J., Buitendam, E., Woodhall, S. C., Hollis, E., Ong, K.-J., Saunders, J., Dunbar, K. and Turner, K. M. E.

Appendix Table 1 Chlamydia retest costs by recall method

Activity	Price per unit (item/ minute of staff time)	Recall method											
		1. Client-led		2. Reminder card		3. SMS invitation		4. Phone invitation		5. Automatic postal test kit		6. Advice at follow-up & SMS	
		Units	Cost	Units	Cost	Units	Cost	Units	Cost	Units	Cost	Units	Cost
**PATIENT OFFERED RETEST**													
Nurse-led conversation about retesting at first diagnosis and issue subsequent reminders													
Nurse band 5/6/7/8[2]	£ 0.89	3	£ 2.68	3	£ 2.68	3	£ 2.68	13	£ 11.60	3	£ 2.68	13	£ 11.60
Blend admin/clerical[1]	£ 0.53	0	£ -	0	£ -	5	£ 2.64	5	£ 2.64	0	£ -	10	£ 5.27
Reminder card	£ 0.10	0	£ -	1	£ 0.10	0	£ -	0	£ -	0	£ -	0	£ -
SMS text message	£ 0.10	0	£ -	0	£ -	1	£ 0.10	0	£ -	0	£ -	1	£ 0.10
Phone call[2]	£ 0.07	0	£ -	0	£ -	0	£ -	3	£ 0.21	0	£ -	3	£ 0.21
**PATIENT DELIVERED RETEST[8]**													
Register, meet and greet													
Blend admin/clerical[3]	£ 0.53	3.8	£ 2.00	3.8	£ 2.00	3.8	£ 2.00	3.8	£ 2.00	0.0	£ -	3.8	£ 2.00
Actual retest													
Consultation - blend of Community SRH staff (N2 to Doctor)[3]	£ 1.06	6.8	£ 7.22	6.8	£ 7.22	6.8	£ 7.22	6.8	£ 7.22	0.0	£ -	6.8	£ 7.22
Blend admin/clerical[1,4]	£ 0.53	1.2	£ 0.63	1.2	£ 0.63	1.2	£ 0.63	1.2	£ 0.63	5	£ 2.64	1.2	£ 0.63
Health professional-led retest - blend of Community SRH staff (N2 to Doctor)[3]	£ 1.06	4.56	£ 4.81	4.56	£ 4.81	4.56	£ 4.81	4.56	£ 4.81	0	£ -	4.56	£ 4.81
Gloves[3]	£ 0.05	0.76	£ 0.03	0.76	£ 0.03	0.76	£ 0.03	0.76	£ 0.03	0	£ -	0.76	£ 0.03
Lab request form with bag	£ 0.10	1	£ 0.10	1	£ 0.10	1	£ 0.10	1	£ 0.10	1	£ 0.10	1	£ 0.10

For peer review only - http://bmjopen.bmj.com/site/about/guidelines.xhtml

Sample collection instructions	£ 0.05	1	£ 0.05	1	£ 0.05	1	£ 0.05	1	£ 0.05	1	£ 0.05	1	£ 0.05
Transport tube	£ 0.26	1	£ 0.26	1	£ 0.26	1	£ 0.26	1	£ 0.26	1	£ 0.26	1	£ 0.26
Urine pot, sterile collection	£ 0.23	0.7	£ 0.16	0.7	£ 0.16	0.7	£ 0.16	0.7	£ 0.16	0.7	£ 0.16	0.7	£ 0.16
Urine specimen container (PCR tube and pipette)	£ 1.04	0.7	£ 0.73	0.7	£ 0.73	0.7	£ 0.73	0.7	£ 0.73	0.7	£ 0.73	0.7	£ 0.73
Vulvo-vaginal swab	£ 0.16	0.3	£ 0.05	0.3	£ 0.05	0.3	£ 0.05	0.3	£ 0.05	0.3	£ 0.05	0.3	£ 0.05
Postage/packaging[4]	£ 0.89	0.24	£ 0.21	0.24	£ 0.21	0.24	£ 0.21	0.24	£ 0.21	1	£ 0.89	0.24	£ 0.21
Return envelope and postage[4]	£ 0.89	0.24	£ 0.21	0.24	£ 0.21	0.24	£ 0.21	0.24	£ 0.21	1	£ 0.89	0.24	£ 0.21
Health promotion/Q&A[6]													
Health professional-led discussion - blend of Community SRH staff (N2 to Doctor)	£ 1.06	6	£ 6.33	6	£ 6.33	6	£ 6.33	6	£ 6.33	6	£ 6.33	6	£ 6.33
KY lubricant	£ 0.30	2	£ 0.60	2	£ 0.60	2	£ 0.60	2	£ 0.60	2	£ 0.60	2	£ 0.60
STI literature	£ 0.06	3	£ 0.18	3	£ 0.18	3	£ 0.18	3	£ 0.18	3	£ 0.18	3	£ 0.18
Male condom	£ 0.06	10	£ 0.58	10	£ 0.58	10	£ 0.58	10	£ 0.58	10	£ 0.58	10	£ 0.58
**RETEST PROCESSED AND RESULTS GIVEN**													
Pathology													
Lab processing	£ 12.51	1	£ 12.51	1	£ 12.51	1	£ 12.51	1	£ 12.51	1	£ 12.51	1	£ 12.51
Results management (retest negatives)													
Nurse band 5/6	£ 0.75	6	£ 4.50	6	£ 4.50	6	£ 4.50	6	£ 4.50	6	£ 4.50	6	£ 4.50
Letter notification	£ 0.58	0.02	£ 0.01	0.02	£ 0.01	0.02	£ 0.01	0.02	£ 0.01	0.02	£ 0.01	0.02	£ 0.01
Phone call	£ 0.07	0.03	£ 0.00	0.03	£ 0.00	0.03	£ 0.00	0.03	£ 0.00	0.03	£ 0.00	0.03	£ 0.00
SMS text message	£ 0.10	0.95	£ 0.10	0.95	£ 0.10	0.95	£ 0.10	0.95	£ 0.10	0.95	£ 0.10	0.95	£ 0.10
Results management (retest positives/equivocal)													
Nurse band 5/6/7/8	£ 0.89	15	£ 13.38	15	£ 13.38	15	£ 13.38	15	£ 13.38	15	£ 13.38	15	£ 13.38
Letter notification	£ 0.58	0.05	£ 0.03	0.05	£ 0.03	0.05	£ 0.03	0.05	£ 0.03	0.05	£ 0.03	0.05	£ 0.03
Phone call	£ 0.07	0.05	£ 0.00	0.05	£ 0.00	0.05	£ 0.00	0.05	£ 0.00	0.05	£ 0.00	0.05	£ 0.00
SMS text message	£ 0.10	0.9	£ 0.09	0.9	£ 0.09	0.9	£ 0.09	0.9	£ 0.09	0.9	£ 0.09	0.9	£ 0.09
Treatment[7]	£ 1.80	1	£ 1.80	1	£ 1.80	1	£ 1.80	1	£ 1.80	1	£ 1.80	1	£ 1.80
Follow-up call[5]													
Blend admin/clerical[1,4]	£ 0.53	5	£ 2.64	5	£ 2.64	5	£ 2.64	5	£ 2.64	5	£ 2.64	5	£ 2.64
Nurse band 5/6[2]	£ 0.75	10	£ 7.50	10	£ 7.50	10	£ 7.50	10	£ 7.50	10	£ 7.50	10	£ 7.50
Phone call[2]	£ 0.07	3	£ 0.21	3	£ 0.21	3	£ 0.21	3	£ 0.21	3	£ 0.21	3	£ 0.21

Shaded entries were taken directly from the basic cost of a (first) chlamydia test[1] which is reproduced here under a Creative Commons licence:

© Pathway Analytics

All other costs are either amended costs from Pathway Analytics (see below for explanation), or costs added in.

[1]Any contact at a distance with client further to the initial retest conversation is assumed to require 5 minutes of admin time to retrieve and update the client's details on a database. [2]A phone call is assumed to be 3 minutes in length but requiring 10 minutes of nurse time to accommodate chasing time. [3]Applicable to clinic retesting: these costs are removed entirely where retesting involves postal testing only, and reduced

proportionally for the remaining recall methods to allow for some clients opting for postal retesting. [4]Applies to postal kits: includes postage to client's address and return postage[2], and associated admin time for sending out a kit. [5]Positives only. [6]It is assumed that all clients will received these at some point including those opting for retesting by post. [7]4 x 250mg of azithromycin in tablet form[3]. VAT, dispensing costs and costs associated with a test for cure are not included. [8]Number of units for clinic vs postal testing kit costs obtained by multiplying base costs by the percentage using each.

For peer review only

Appendix Figure 1 Retesting pathway

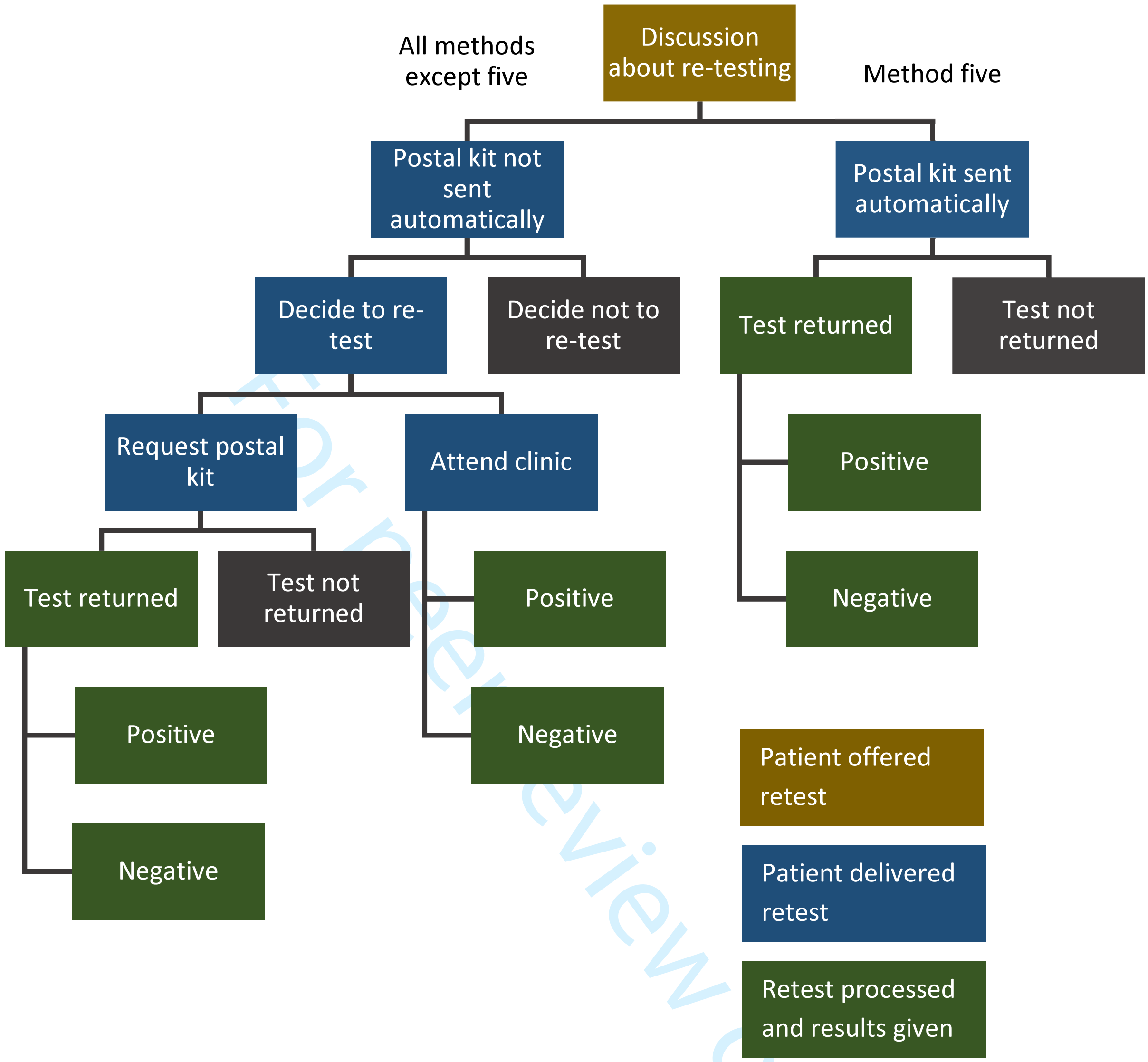

For peer review only

Appendix Table 2 Parameter values

Parameter	Baseline value (10-14 weeks since treatment for first infection)		Sensitivity value (10-26 weeks since treatment for first infection)		Reference
Retest uptake (i.e., percentage of (first) positives who choose to accept retesting) (all methods except method five)[1]	1. Client-led	5%	1. Client-led	15%	NCSP audit report[4] and NCSP audit data provided by PHE
	2. Reminder card	4%	2. Reminder card	19%	
	3. SMS invitation	9%	3. SMS invitation	21%	
	4. Phone invitation	7%	4. Phone invitation	17%	
	6. Advice at follow-up & SMS	13%	6. Advice at follow-up & SMS	25%	
Percentage of those retesting who choose to attend a clinic for a retest (all methods except method five)[2]	76%		73%		NCSP audit data provided by PHE
Percentage of those retesting who choose to request a postal kit (all methods except method five)	=100-76% =24%		=100-73% =27%		
Postal test kit return rate (requested kits)	67%		67%		Retesting pilot[5]
Postal test kit return rate (kits sent out automatically; method five)	5. Automatic postal test kit	10%	5. Automatic postal test kit	23%	NCSP audit report[4] and NCSP audit data provided by PHE
Chlamydia retest positivity[3]	12%		16%		NCSP audit report[4] and NCSP audit data provided by PHE

[1]Obtained by fitting to overall retest rates from the audit (i.e., accounting for non-return of requested postal kits); [2]The audit only has data on percentage of *completed* retests obtained from clinic testing vs postal testing (for those instances where a kit was not sent out automatically), not percentage of those who opt for a retest at a clinic among *all* retesters (i.e., including all those who request a kit, some of whom do not return the kit); [3]Average over the six most commonly-used methods.

References

1. Pathway Analytics: Pathway Prices for Integrated Sexual Health Tariffs *118. T3 Chlamydia, gonorrhoea and syphilis tests* *https://www.pathwayanalytics.com/pathways/26-t3-chlamydia-gonorrhoea-syphilis-tests/118-t3-chlamydia-gonorrhoea-and-syphilis-tests* *Accessed 2013*.
2. *Royal Mail: Get a price http://www.royalmail.com/price-finder Accessed 14/08/2015*.
3. *British National Formulary: Azithromycin http://www.evidence.nhs.uk/formulary/bnf/current/5-infections/51-antibacterial-drugs/515-macrolides/azithromycin Accessed 02/02/2016*.
4. *Re-testing of those who tested positive for chlamydia: National audit report https://www.gov.uk/government/uploads/system/uploads/attachment_data/file/471585/NCSPre-testingauditfinalversion.pdf Accessed 13/09/2016*.
5. Angel, G., et al., *An observational study to evaluate three pilot programmes of retesting chlamydia-positive individuals within 6 months in the South West of England.* BMJ Open, 2016. **5**(10): p. e007455.

For peer review only

CHEERS checklist—Items to include when reporting economic evaluations of health interventions (BMJ 2013;346:f1049)

Section/item	Item No	Recommendation	Reported on page No/ lin No
**Title and abstract**			
Title	1	Identify the study as an economic evaluation or use more specific terms such as "cost-effectiveness analysis", and describe the interventions compared.	p1/line 1-3
Abstract	2	Provide a structured summary of objectives, perspective, setting, methods (including study design and inputs), results (including base case and uncertainty analyses), and conclusions.	p2
**Introduction**			
Background and objectives	3	Provide an explicit statement of the broader context for the study.	p3/line 67-73
		Present the study question and its relevance for health policy or practice decisions.	p3-4/line 90-95
**Methods**			
Target population and subgroups	4	Describe characteristics of the base case population and subgroups analysed, including why they were chosen.	N/A
Setting and location	5	State relevant aspects of the system(s) in which the decision(s) need(s) to be made.	p4/line 97
Study perspective	6	Describe the perspective of the study and relate this to the costs being evaluated.	p4/line 97-98
Comparators	7	Describe the interventions or strategies being compared and state why they were chosen.	p4/line 97-98

For peer review only - http://bmjopen.bmj.com/site/about/guidelines.xhtml

Section/item	Item No	Recommendation	Reported on page No/ line No
Time horizon	8	State the time horizon(s) over which costs and consequences are being evaluated and say why appropriate.	p4/line 97-100
Discount rate	9	Report the choice of discount rate(s) used for costs and outcomes and say why appropriate.	N/A
Choice of health outcomes	10	Describe what outcomes were used as the measure(s) of benefit in the evaluation and their relevance for the type of analysis performed.	N/A
Measurement of effectiveness	11a	*Single study-based estimates:* Describe fully the design features of the single effectiveness study and why the single study was a sufficient source of clinical effectiveness data.	N/A
	11b	*Synthesis-based estimates:* Describe fully the methods used for identification of included studies and synthesis of clinical effectiveness data.	N/A
Measurement and valuation of preference based outcomes	12	If applicable, describe the population and methods used to elicit preferences for outcomes.	N/A
Estimating resources and costs	13a	*Single study-based economic evaluation:* Describe approaches used to estimate resource use associated with the alternative interventions. Describe primary or secondary research methods for valuing each resource item in terms of its unit cost. Describe any adjustments made to approximate to opportunity costs.	p4/line 99-104 and Appendix Table 1
	13b	*Model-based economic evaluation:* Describe approaches and data sources used to estimate resource use associated with model health states.	N/A

Section/item	Item No	Recommendation	Reported on page No/ line No
		Describe primary or secondary research methods for valuing each resource item in terms of its unit cost. Describe any adjustments made to approximate to opportunity costs.	
Currency, price date, and conversion	14	Report the dates of the estimated resource quantities and unit costs. Describe methods for adjusting estimated unit costs to the year of reported costs if necessary. Describe methods for converting costs into a common currency base and the exchange rate.	pP4/line 100
Choice of model	15	Describe and give reasons for the specific type of decision-analytical model used. Providing a figure to show model structure is strongly recommended.	Appendix Figure 1
Assumptions	16	Describe all structural or other assumptions underpinning the decision-analytical model.	p4/line 107-120 and Appendix Table 2
Analytical methods	17	Describe all analytical methods supporting the evaluation. This could include methods for dealing with skewed, missing, or censored data; extrapolation methods; methods for pooling data; approaches to validate or make adjustments (such as half cycle corrections) to a model; and methods for handling population heterogeneity and uncertainty.	N/A
**Results**			
Study parameters	18	Report the values, ranges, references, and, if used, probability distributions for all parameters. Report reasons or sources for distributions used to represent uncertainty where appropriate. Providing a table to show the input values is strongly recommended.	p4/line 107-120 and Appendix Table 2

Section/item	Item No	Recommendation	Reported on page No/ line No
Incremental costs and outcomes	19	For each intervention, report mean values for the main categories of estimated costs and outcomes of interest, as well as mean differences between the comparator groups. If applicable, report incremental cost-effectiveness ratios.	Table 1
Characterising uncertainty	20a	*Single study-based economic evaluation:* Describe the effects of sampling uncertainty for the estimated incremental cost and incremental effectiveness parameters, together with the impact of methodological assumptions (such as discount rate, study perspective).	N/A
	20b	*Model-based economic evaluation:* Describe the effects on the results of uncertainty for all input parameters, and uncertainty related to the structure of the model and assumptions.	Table 1
Characterising heterogeneity	21	If applicable, report differences in costs, outcomes, or cost-effectiveness that can be explained by variations between subgroups of patients with different baseline characteristics or other observed variability in effects that are not reducible by more information.	N/A
**Discussion**			
Study findings, limitations, generalisability, and current knowledge	22	Summarise key study findings and describe how they support the conclusions reached. Discuss limitations and the generalisability of the findings and how the findings fit with current knowledge.	p7-8
**Other**			

For peer review only - http://bmjopen.bmj.com/site/about/guidelines.xhtml

Section/item	Item No	Recommendation	Reported on page No/ line No
Source of funding	23	Describe how the study was funded and the role of the funder in the identification, design, conduct, and reporting of the analysis. Describe other non-monetary sources of support.	p8-9
Conflicts of interest	24	Describe any potential for conflict of interest of study contributors in accordance with journal policy. In the absence of a journal policy, we recommend authors comply with International Committee of Medical Journal Editors recommendations.	p9

For consistency, the CHEERS statement checklist format is based on the format of the CONSORT statement checklist

For peer review only

BMJ Open

An economic evaluation of the cost of different methods of retesting chlamydia positive individuals in England

Journal:	*BMJ Open*
Manuscript ID	bmjopen-2018-024828.R2
Article Type:	Research
Date Submitted by the Author:	11-Jan-2019
Complete List of Authors:	Looker, Katharine; University of Bristol, Population Health Sciences, Bristol Medical School<br>Buitendam, Erna; Public Health England, HIV & STI Department, National Infection Service<br>Woodhall, Sarah; Public Health England, HIV & STI; UCL, Research Department of Infection and Population Health<br>Hollis, Emma; Public Health England, HIV/STI<br>Ong, Koh Jun; Public Health England, National Infections Service - HIV/STI<br>Saunders, John; Public Health England, HIV & STI Department; University College London, Research Department of Infection and Population Health<br>Dunbar, Kevin; Public Health England, HIV & STI Department<br>Turner, Katy; Bristol University, School of Social and Community Medicine
**Primary Subject Heading**:	Sexual health
Secondary Subject Heading:	Health services research, Health economics, Public health, Infectious diseases
Keywords:	Public health < INFECTIOUS DISEASES, HEALTH SERVICES ADMINISTRATION & MANAGEMENT, HEALTH ECONOMICS

SCHOLARONE™
Manuscripts

An economic evaluation of the cost of different methods of retesting chlamydia positive individuals in England

Looker, K. J.[1]*, Buitendam, E.[2], Woodhall, S. C.[2], Hollis, E.[2], Ong, K.-J.[2], Saunders, J.[2], Dunbar, K.[2] and Turner, K. M. E.[1]

[1]Population Health Sciences, Bristol Medical School, University of Bristol, Bristol, United Kingdom

[2]HIV & STI Department, National Infection Service, Public Health England, United Kingdom

*Corresponding author. Correspondence should be sent to:

Dr Katharine Looker

Population Health Sciences, Bristol Medical School

University of Bristol

Oakfield House, Oakfield Grove

Bristol BS8 2BN

United Kingdom

katharine.looker@bristol.ac.uk

For peer review only

For peer review only - http://bmjopen.bmj.com/site/about/guidelines.xhtml

ABSTRACT

Objectives

The National Chlamydia Screening Programme in England opportunistically screens eligible individuals for chlamydia infection. Retesting is recommended three months after treatment following a positive test result, but no guidance is given on how local areas should recall individuals for retesting. Here we compare cost estimates for different recall methods to inform the optimal delivery of retesting programmes.

Design

Economic evaluation

Setting

England

Methods

We estimated the cost of chlamydia retesting for each of the six most commonly-used recall methods in 2014 based on existing cost estimates of a chlamydia screen. Proportions accepting retesting, opting for retesting by post, returning postal testing kits and retesting positive were informed by 2014 NCSP audit data. Health professionals "sense-checked" the costs.

Primary and secondary outcomes

Cost and adjusted cost per chlamydia retest; cost and adjusted cost per chlamydia retest positive.

Results

We estimated the cost of the chlamydia retest pathway, including treatment/follow-up call, to be between £45-£70 per completed test. At the lower end this compared favourably to the cost of a clinic-based screen. Cost per retest positive was £389-£607. After adjusting for incomplete uptake, and non-return of postal kits, the cost rose to £109-£289 per completed test (cost per retest positive: £946-£2,506). The most economical method in terms of adjusted cost per retest was no active recall, as gains in retest rates with active recall did not outweigh the higher cost. Nurse-led client contact by phone was particularly uneconomical, as was sending out postal testing kits automatically.

Conclusions

Retesting without active recall is more economical than more intensive methods such as recalling by phone and automatically sending out postal kits. If sending an SMS could be automated, this could be the most economical way of delivering retesting. However, patient choice and local accessibility of services should be taken into consideration in planning.

For peer review only - http://bmjopen.bmj.com/site/about/guidelines.xhtml

For peer review only

Strengths and limitations of this study

We compared the cost of the chlamydia retest pathway in England across the five most commonly-used methods of recalling individuals for retesting, to enable local service planners to assess whether they are delivering retesting economically or should consider an alternative approach.

Our cost estimates included both clinic retesting, and retesting using postal kits.

We incorporated incomplete uptake, and non-return of postal kits, to estimate cost based on actual patterns of use.

We did not specifically look at the effect of factors such as gender, country of birth, sexual orientation, perceived risk of infection and presence of symptoms on retest uptake and therefore cost, although no active recall is likely to be associated with similar or higher test positivity than active recall.

We also did not consider other important factors besides cost such as the demography of the population: for example, automatically sending out postal kits might be the only feasible option in rural areas, and indeed, on-line testing, which was not considered in our analysis, is likely to be the most economical method of all.

INTRODUCTION

Chlamydia trachomatis (chlamydia) is the most commonly-diagnosed bacterial sexually-transmitted infection (STI) in England[1]. Since 2003 there has been a National Chlamydia Screening Programme (NCSP) in England which opportunistically tests sexually-active 15-24 year olds[2]. NCSP guidelines recommend retesting three months after treatment for chlamydia[3]. British Association for Sexual Health and HIV (BASHH) national guidelines recommend retesting under 25 year olds three to six months after treatment[4]. No guidance is given by either the NCSP or BASHH on how local areas should recall individuals for retesting, which can be done in many ways. The 2014 NCSP retesting audit[5] found that the most common methods of recalling individuals for retesting were as follows: (1) conversation with client at time of test result with no further reminder (32%); (2) reminder card given to client at time of test result with no further reminder (1%); (3) client sent text message when retest due (29%); (4) client invited by phone call when retest due (8%); (5) testing kit posted to client's chosen address when retest due (5%); and (6) retesting advised at follow up call with client - text message sent at 3 months (19%). The audit also measured overall retest rates for each recall method, which were calculated from the number that attended a clinic for a retest or returned a postal testing kit, divided by the total number recalled for retesting. This is different to retest uptake, which is the number that attended a clinic for a retest or *ordered* or were sent a postal testing kit, divided by the total number recalled for retesting, which has cost implications. Retest uptake was not measured by the audit. Unpublished data from the 2017 NCSP retesting audit (Erna Buitendam, personal communication) showed that, for the six most commonly-used recall methods in the 2014 audit, retest rates significantly increased for method one (client-led) and method five (automatic postal test kit) between the 2014 and 2017 audits.

Previous estimates exist for the cost of a clinic-based chlamydia screen[6, 7]. However, to our knowledge there are no estimates of the cost of a chlamydia retest, and how this varies by recall

For peer review only - http://bmjopen.bmj.com/site/about/guidelines.xhtml

method. Specifically, we do not know the best way to balance getting the optimal number of people to retest versus the additional cost of delivering invitations or reminders to retest. Understanding how the cost of retesting varies depending on the approach taken is critical for optimal programme delivery. Here we present cost estimates for different recall methods in England, firstly for the retest pathway itself, and then for the adjusted cost per retest, allowing for incomplete uptake, and non-return of postal kits, to impact on cost.

METHODS

We estimated the cost of chlamydia retesting in England using Microsoft Excel 2016 for each of the six most commonly-used recall methods reported in the 2014 NCSP retesting audit[5] (Table 1) as follows. First, we entered existing cost estimates for a chlamydia test from Pathway Analytics (costed for clinic-based chlamydia screening for 2011), which excluding a follow-up call was around £45[6] (Appendix Table 1). We used this costing as given. We then added additional costs to reflect costs specifically associated with retesting, such as a nurse-led conversation about retesting after diagnosis, and issuing retest invitations/reminders (e.g., by text message [SMS] or phone). A nurse-led conversation about retesting after diagnosis was assumed to involve only extra nurse time to additionally discuss retesting; other associated costs were assumed to be already factored into the cost of a first test. Both an SMS and a phone call were assumed to involve administration time and the cost of the SMS or call itself, while the latter was assumed to also include both nurse time for the actual call as well as chasing time. In addition, we amended the clinic-based chlamydia test costs to allow for postal testing. Costs were then totalled across the following cost categories: cost of offering retesting, cost of delivering retest, and cost of processing retest and giving results, as well as overall.

For each of the six recall methods, we costed both the retest pathway, and the adjusted cost per retest (Appendix Figure 1). The adjusted cost per retest accounts for incomplete uptake, and non-return of postal kits, within each cost category. For all methods except method five (automatic postal testing kit) we allowed clients to choose either to attend a clinic for retesting, or to request a postal testing kit. Thus, for methods one to four, and method six, we incorporated the following parameters: retest uptake, the proportion who opt for postal testing, and the return rate of requested kits. Retest uptake for each of the six recall methods was fitted to overall retest rates from the 2014 NCSP audit[5], taking a value of 24% for the proportion of clients who opt for postal testing (also from the audit), and a value of 67% for the return rate of requested kits[8]. For method five, uptake was equivalent to overall retest rate and was simply the return rate of postal kits (10%) from the 2014 NCSP audit[5]. Chlamydia retest positivity (12%), which informs the relative weight given to the cost of managing a positive retest result versus managing a negative retest result in the average cost of the chlamydia retesting pathway, was taken from the NCSP audit[5], and was averaged over all six recall methods due to small numbers by individual method. We also calculated the cost and adjusted cost per retest positive, i.e., the cost of finding one positive retest incorporating the cost of other, negative retests, by dividing test costs by the chlamydia positivity. For a table of parameter values see Appendix Table 2.

The time frame for calculating the parameter values was 10-14 weeks, corresponding to NCSP guidance for retesting. We sense-checked our retesting costs (Appendix Table 1) with health professionals. We conducted two sensitivity analyses. In the first sensitivity analysis we replaced the parameters for the retesting pathway with those obtained from data for retesting done between 10-26 weeks (corresponding to BASHH guidance) (Appendix Table 2). This simply allows more time for

For peer review only - http://bmjopen.bmj.com/site/about/guidelines.xhtml

clients to retest: there is no additional contact with clients to remind them to retest. In the second sensitivity analysis we altered staff salary costs from nurse bands to administrator bands for nurse-based costs associated with phone invitations to retest, managing a retest negative, and a follow-up call at three months for those retesting positive (leaving the nurse-based costs associated with the initial retest conversation and managing a retest positive unchanged). The purpose of this was to show the difference in price that could be achieved if administrative staff instead of nurses contacted clients by phone, except where a lower band of advisor might not be appropriate.

Since retest rates significantly increased for method one (client-led) and method five (automatic postal test kit) between the 2014 and (unpublished) 2017 audits (p>0.05), we restricted our analyses to 2014 data only. However, we carried out an analysis of whether retest positivity was statistically-significantly different for no active recall (method one) versus active recall (methods three and six) using both 2014 and 2017 audit data, since there was no statistically-significant difference in the positivity rates for each of these methods when comparing 2014 and 2017 data.

Patient and Public Involvement

Patients and the public were not involved in this analysis.

For peer review only

For peer review only - http://bmjopen.bmj.com/site/about/guidelines.xhtml

Table 1 Chlamydia retest costs by recall method

	Recall method					
	1. Client-led	2. Reminder card	3. SMS invitation	4. Phone invitation	5. Automatic postal test kit	6. Advice at follow-up & SMS
Number of retest invitations by each method (%), N=2853[1] (NCSP audit, 2014[5])	**912 (32%)**	**27 (1%)**	**840 (29%)**	**227 (8%)**	**130 (5%)**	**528 (19%)**
Description	Conversation with client at time of test result with no further reminder	Reminder card given to client at time of test result with no further reminder	Client sent text message when retest due	Client invited by phone call when retest due	Testing kit posted to client's chosen address when retest due	Retesting advised at follow up call with client - text message sent at 3 months
**COSTS USING BASELINE PARAMETERS (10-14 weeks since treatment for first infection)**						
**Cost of chlamydia retesting pathway[2]**						
Cost of offering retesting	**£2.68**	**£2.78**	**£5.42**	**£14.44**	**£2.68**	**£17.18**
Cost of delivering retest	**£24.16**	**£24.16**	**£24.16**	**£24.16**	**£13.45**	**£24.16**
Cost of processing retest and giving results	**£28.71**	**£28.71**	**£28.71**	**£28.71**	**£28.71**	**£28.71**
**TOTAL COST**	**£55.54**	**£55.64**	**£58.28**	**£67.31**	**£44.83**	**£70.05**
**Cost per retest positive**	**£481**	**£482**	**£505**	**£583**	**£389**	**£607**
**Retest uptake**	**5%**	**4%**	**9%**	**7%**	**10%**	**13%**
**Retest rate**	**5%**	**4%**	**8%**	**6%**	**10%**	**12%**
**Adjusted cost per retest incorporating incomplete uptake/non-return of kits**	**£109**	**£130**	**£120**	**£289**	**£190**	**£195**
**Adjusted cost per retest positive incorporating incomplete uptake/non-return of kits**	**£946**	**£1,126**	**£1,039**	**£2,506**	**£1,646**	**£1,686**
**COSTS USING LONGER TIME WINDOW FOR RETESTING (10-26 weeks since treatment for first infection)**						
**Total cost of chlamydia retesting pathway**	**£55.38**	**£55.48**	**£58.12**	**£67.15**	**£45.32**	**£69.89**
**Cost per retest positive**	**£344**	**£345**	**£361**	**£417**	**£282**	**£435**
**Retest uptake**	**16%**	**20%**	**23%**	**18%**	**23%**	**27%**
**Retest rate**	**15%**	**19%**	**21%**	**17%**	**23%**	**25%**
**Adjusted cost per retest incorporating incomplete uptake/non-return of kits**	**£73**	**£71**	**£82**	**£142**	**£99**	**£126**
**Adjusted cost per retest positive incorporating incomplete uptake/non-return of kits**	**£456**	**£440**	**£508**	**£883**	**£616**	**£780**
**COSTS IF ADMINISTRATORS USED INSTEAD OF NURSES (10-14 weeks since treatment for first infection)**						
**Total cost of chlamydia retesting pathway**	**£52.13**	**£52.23**	**£54.87**	**£60.24**	**£41.42**	**£62.98**
**Cost per retest positive**	**£452**	**£453**	**£476**	**£522**	**£359**	**£546**
**Adjusted cost per retest incorporating incomplete uptake/non-return of kits**	**£106**	**£126**	**£117**	**£227**	**£187**	**£161**
**Adjusted cost per retest positive incorporating incomplete uptake/non-return of kits**	**£917**	**£1,096**	**£1,010**	**£1,963**	**£1,617**	**£1,399**

[1]Other methods or method not recorded account for the remaining 7% (N=189) of retests. [2]Some costs were taken (and some subsequently amended) from the basic cost of a (first) chlamydia test[6] which is under a Creative Commons licence:

For peer review only - http://bmjopen.bmj.com/site/about/guidelines.xhtml

© Pathway Analytics

RESULTS

The estimated cost of the chlamydia retest pathway ranged from £45-£70 per completed test, while the cost per retest positive ranged from £389-£607 (Table 1). Posting testing kits automatically with no further reminder (method five) was the cheapest recall method, while methods involving inviting clients by phone to retest (methods four and six) were the most expensive. After adjusting for incomplete uptake and non-return of postal kits, the cost per chlamydia retest ranged from £109-£289 per completed test, while the cost per retest positive ranged from £946-£2,506. Here, the most economical recall method in terms of the adjusted cost per retest was no active recall (method one). An SMS invitation (method three) increased retest rates for comparatively small additional cost. The most expensive methods were still those involving inviting clients by phone to retest (methods four and six). This was despite these methods achieving higher retest rates (6% and 12% for methods four and six, respectively) compared to no active recall (5%). Sending postal testing kits out automatically (method five) was also an uneconomical way of delivering a retest, due to the cost of non-returned kits. Retest positivity was not statistically-significantly different for no active recall (method one) versus active recall (methods three and six) when 2014 and 2017 audit data were combined.

Extending the retesting period to 10-26 weeks did not impact substantially on the chlamydia retesting pathway cost (range £45-£70) (Table 1). However, the adjusted cost per retest incorporating incomplete uptake and non-return of kits was substantially lower (range £71-£126), as was the adjusted cost per retest positive (range £440-£883), than with a tighter retest window, particularly for automatically sending out postal kits (method five). However, this assumed positivity was higher for the 10-26 week window across all methods. In any case, methods with no or else minimal active recall were still the most economical. Replacing nurse bands with administrator bands only had a substantial impact on costs for those methods where clients were contacted by phone to recall for retest (Table 1).

DISCUSSION

The estimated cost of the chlamydia retest pathway ranged from £45-£70 per completed test, which at the cheapest end was very similar to the cost of a clinic-based chlamydia screen[6, 7]. The cost per retest positive, meanwhile, ranged from £389-£607. Important differences were seen when uptake and kit return rates were varied. This is because successfully completed retests effectively absorbed the cost of incomplete retests. Here, the most economical recall method involved no active recall after the initial retest conversation. Sending out postal testing kits automatically was an expensive way of doing retesting because of wastage of kits. However, the most expensive methods involved contacting clients by phone to invite them to retest, primarily because of nurse time required.

When the retesting window was increased from 10-14 weeks to 10-26 weeks, all methods of recall had a reduced adjusted cost per retest, due in part to higher positivity for 10-26 weeks. However, a longer time window means there is further potential for onward transmission, so it is important clients are counselled on the best time to retest.

For peer review only - http://bmjopen.bmj.com/site/about/guidelines.xhtml

Active recall increased retest rates but this did not outweigh the additional cost. We assumed that sending an SMS involved administration time to retrieve clients' details from a database. In our analysis we considered only the effect of altering staff salary costs from nurse bands to administrator bands for some nurse-based activities. If the time needed to send an SMS could be shortened by automating this process, then an SMS invitation or reminder could be an economical way of increasing retest rates. For example, if the cost of associated administration time is removed, then the adjusted cost per chlamydia retest is £88 and the adjusted cost per retest positive is £765, making sending an SMS the most economical way of delivering retesting. Conversations with health professionals during the course of our study suggested that a shorter administration time to send an SMS was theoretically feasible. We did not find any evidence that retest positivity was different for active recall versus no active recall, meaning there is no evidence that active recall merely results in more negatives being tested. However, evidence from a retesting pilot in South-West England did show that those who retested without being actively recalled had higher chlamydia retest positivity than those who were actively recalled[8]. Furthermore, the unpublished 2017 audit data showed a statistically-significant increase in the retest rate for client-led retesting for 10-14 weeks compared to 2014 (Erna Buitendam, personal communication), which could make no active recall even more economical than shown here.

Our analysis was done for the pathway cost of testing for chlamydia alone[6]. Where chlamydia testing is done at the same time as testing for other STIs (such as gonorrhoea), the proportionate cost of testing for chlamydia will be reduced. Another consideration is that since our analysis was carried out, the estimated pathway cost has fallen, which will reduce costs further across all methods of retesting. However, cost is not the only important factor to consider. For example, no active recall also had the lowest retest rate, although as noted above, active recall may not necessarily identify more infected people if those opting to retest self-select on the basis of their perceived risk or presence of symptoms. We also did not account for the effect on retest uptake of factors such as gender, location of services, country of birth, and sexual orientation. The composition of the population is an important consideration in local planning: a large rural population, for example, might affect how retesting needs to be delivered. Given the much higher return rate for requested postal testing kits compared to kits sent out automatically, online testing with automated recall is likely to be the most economical method of all, but was beyond the scope of this analysis.

Our analysis suggests that no active recall after the initial retest conversation is the most economical way of retesting, although an SMS invitation or reminder could be considered. Patient choice and accessibility of services should still be taken into consideration for local delivery planning and it is important that retest uptake is monitored as this determines how economical retesting is.

CONTRIBUTORS

KJL undertook the itemisation and costing, analysed the results and drafted the manuscript. KMET oversaw the study and provided advice as needed. EB and SW provided audit data and advised on parameterisation. K-JO helped with sources for costs. KJL, EB, SW, EH, K-JO, JS, KD and KMET all contributed to the progress of the study and manuscript revisions.

CONFLICT OF INTEREST

The authors declare there are no conflicts of interest.

For peer review only - http://bmjopen.bmj.com/site/about/guidelines.xhtml

ACKNOWLEDGEMENTS

KJL and KMET thank the National Institute for Health Research (NIHR) Health Protection Research Unit (HPRU) in Evaluation of Interventions at the University of Bristol, in partnership with Public Health England, for research support. We would like to thank Rose Tobin (North East and North Central London Adult Critical Care Network, Royal Free London NHS Foundation Trust), Stephanie Rumsey (East Cheshire NHS Trust), Jan Cremer (Essex Partnership University NHS Foundation Trust) and Stephanie Sawyer (London Borough of Bromley Public Health) for sense-checking the costs, and Pathway Analytics for data on the cost of a chlamydia test.

FUNDING

The research was funded by the National Institute for Health Research Health Protection Research Unit (NIHR HPRU) in Evaluation of Interventions at the University of Bristol in partnership with Public Health England (PHE). The views expressed are those of the author(s) and not necessarily those of the NHS, the NIHR,the Department of Health and Social Care or Public Health England.

DATA AVAILABILITY

All data relevant to the study are included in the article or uploaded as supplementary information.

REFERENCES

1. *National chlamydia screening programme (NCSP): data tables* *https://www.gov.uk/government/statistics/national-chlamydia-screening-programme-ncsp-data-tables* *Accessed 13/09/2016*.
2. *NCSP: programme overview* *https://www.gov.uk/government/publications/ncsp-programme-overview* *Accessed 13/09/2016*.
3. *Consultation Report: Routine offer of re-test to young adults testing positive for chlamydia (Consultation findings and evidence summary)* *https://www.gov.uk/government/uploads/system/uploads/attachment_data/file/508364/NCSP_Report_Consultation_on_Re-testing_of_Positive_Chlamydia_Cases_August_2013_FINAL.pdf* *Accessed 14/09/2016*.
4. Nwokolo, N.C., et al. *2015 UK national guideline for the management of infection with Chlamydia trachomatis* *http://www.bashh.org/documents/UK%20Chlamydia%20Guidelines%202015.pdf* *Accessed 14/09/2016*.
5. *Re-testing of those who tested positive for chlamydia: National audit report* *https://www.gov.uk/government/uploads/system/uploads/attachment_data/file/471585/NCSPre-testingauditfinalversion.pdf* *Accessed 13/09/2016*.
6. Pathway Analytics: Pathway Prices for Integrated Sexual Health Tariffs *118. T3 Chlamydia, gonorrhoea and syphilis tests* *https://www.pathwayanalytics.com/pathways/26-t3-chlamydia-gonorrhoea-syphilis-tests/118-t3-chlamydia-gonorrhoea-and-syphilis-tests* *Accessed 2013*.
7. Turner, K., et al., *Costs and cost effectiveness of different strategies for chlamydia screening and partner notification: an economic and mathematical modelling study.* BMJ, 2011. **342**: p. c7250.
8. Angel, G., et al., *An observational study to evaluate three pilot programmes of retesting chlamydia-positive individuals within 6 months in the South West of England.* BMJ Open, 2016. **5**(10): p. e007455.

For peer review only - http://bmjopen.bmj.com/site/about/guidelines.xhtml

For peer review only

For peer review only - http://bmjopen.bmj.com/site/about/guidelines.xhtml

Appendix

An economic evaluation of the cost of different methods of retesting chlamydia positive individuals in England

Looker, K. J., Buitendam, E., Woodhall, S. C., Hollis, E., Ong, K.-J., Saunders, J., Dunbar, K. and Turner, K. M. E.

Appendix Table 1 Chlamydia retest costs by recall method

Activity	Price per unit (item/ minute of staff time)	Recall method											
		1. Client-led		2. Reminder card		3. SMS invitation		4. Phone invitation		5. Automatic postal test kit		6. Advice at follow-up & SMS	
		Units	Cost	Units	Cost	Units	Cost	Units	Cost	Units	Cost	Units	Cost
**PATIENT OFFERED RETEST**													
Nurse-led conversation about retesting at first diagnosis and issue subsequent reminders													
Nurse band 5/6/7/8[2]	£ 0.89	3	£ 2.68	3	£ 2.68	3	£ 2.68	13	£ 11.60	3	£ 2.68	13	£ 11.60
Blend admin/clerical[1]	£ 0.53	0	£ -	0	£ -	5	£ 2.64	5	£ 2.64	0	£ -	10	£ 5.27
Reminder card	£ 0.10	0	£ -	1	£ 0.10	0	£ -	0	£ -	0	£ -	0	£ -
SMS text message	£ 0.10	0	£ -	0	£ -	1	£ 0.10	0	£ -	0	£ -	1	£ 0.10
Phone call[2]	£ 0.07	0	£ -	0	£ -	0	£ -	3	£ 0.21	0	£ -	3	£ 0.21
**PATIENT DELIVERED RETEST[8]**													
Register, meet and greet													
Blend admin/clerical[3]	£ 0.53	3.8	£ 2.00	3.8	£ 2.00	3.8	£ 2.00	3.8	£ 2.00	0.0	£ -	3.8	£ 2.00
Actual retest													
Consultation - blend of Community SRH staff (N2 to Doctor)[3]	£ 1.06	6.8	£ 7.22	6.8	£ 7.22	6.8	£ 7.22	6.8	£ 7.22	0.0	£ -	6.8	£ 7.22
Blend admin/clerical[1,4]	£ 0.53	1.2	£ 0.63	1.2	£ 0.63	1.2	£ 0.63	1.2	£ 0.63	5	£ 2.64	1.2	£ 0.63
Health professional-led retest - blend of Community SRH staff (N2 to Doctor)[3]	£ 1.06	4.56	£ 4.81	4.56	£ 4.81	4.56	£ 4.81	4.56	£ 4.81	0	£ -	4.56	£ 4.81
Gloves[3]	£ 0.05	0.76	£ 0.03	0.76	£ 0.03	0.76	£ 0.03	0.76	£ 0.03	0	£ -	0.76	£ 0.03
Lab request form with bag	£ 0.10	1	£ 0.10	1	£ 0.10	1	£ 0.10	1	£ 0.10	1	£ 0.10	1	£ 0.10

For peer review only

Sample collection instructions	£ 0.05	1	£ 0.05	1	£ 0.05	1	£ 0.05	1	£ 0.05	1	£ 0.05	1	£ 0.05
Transport tube	£ 0.26	1	£ 0.26	1	£ 0.26	1	£ 0.26	1	£ 0.26	1	£ 0.26	1	£ 0.26
Urine pot, sterile collection	£ 0.23	0.7	£ 0.16	0.7	£ 0.16	0.7	£ 0.16	0.7	£ 0.16	0.7	£ 0.16	0.7	£ 0.16
Urine specimen container (PCR tube and pipette)	£ 1.04	0.7	£ 0.73	0.7	£ 0.73	0.7	£ 0.73	0.7	£ 0.73	0.7	£ 0.73	0.7	£ 0.73
Vulvo-vaginal swab	£ 0.16	0.3	£ 0.05	0.3	£ 0.05	0.3	£ 0.05	0.3	£ 0.05	0.3	£ 0.05	0.3	£ 0.05
Postage/packaging[4]	£ 0.89	0.24	£ 0.21	0.24	£ 0.21	0.24	£ 0.21	0.24	£ 0.21	1	£ 0.89	0.24	£ 0.21
Return envelope and postage[4]	£ 0.89	0.24	£ 0.21	0.24	£ 0.21	0.24	£ 0.21	0.24	£ 0.21	1	£ 0.89	0.24	£ 0.21
Health promotion/Q&A[6]													
Health professional-led discussion - blend of Community SRH staff (N2 to Doctor)	£ 1.06	6	£ 6.33	6	£ 6.33	6	£ 6.33	6	£ 6.33	6	£ 6.33	6	£ 6.33
KY lubricant	£ 0.30	2	£ 0.60	2	£ 0.60	2	£ 0.60	2	£ 0.60	2	£ 0.60	2	£ 0.60
STI literature	£ 0.06	3	£ 0.18	3	£ 0.18	3	£ 0.18	3	£ 0.18	3	£ 0.18	3	£ 0.18
Male condom	£ 0.06	10	£ 0.58	10	£ 0.58	10	£ 0.58	10	£ 0.58	10	£ 0.58	10	£ 0.58
**RETEST PROCESSED AND RESULTS GIVEN**													
Pathology													
Lab processing	£ 12.51	1	£ 12.51	1	£ 12.51	1	£ 12.51	1	£ 12.51	1	£ 12.51	1	£ 12.51
Results management (retest negatives)													
Nurse band 5/6	£ 0.75	6	£ 4.50	6	£ 4.50	6	£ 4.50	6	£ 4.50	6	£ 4.50	6	£ 4.50
Letter notification	£ 0.58	0.02	£ 0.01	0.02	£ 0.01	0.02	£ 0.01	0.02	£ 0.01	0.02	£ 0.01	0.02	£ 0.01
Phone call	£ 0.07	0.03	£ 0.00	0.03	£ 0.00	0.03	£ 0.00	0.03	£ 0.00	0.03	£ 0.00	0.03	£ 0.00
SMS text message	£ 0.10	0.95	£ 0.10	0.95	£ 0.10	0.95	£ 0.10	0.95	£ 0.10	0.95	£ 0.10	0.95	£ 0.10
Results management (retest positives/equivocal)													
Nurse band 5/6/7/8	£ 0.89	15	£ 13.38	15	£ 13.38	15	£ 13.38	15	£ 13.38	15	£ 13.38	15	£ 13.38
Letter notification	£ 0.58	0.05	£ 0.03	0.05	£ 0.03	0.05	£ 0.03	0.05	£ 0.03	0.05	£ 0.03	0.05	£ 0.03
Phone call	£ 0.07	0.05	£ 0.00	0.05	£ 0.00	0.05	£ 0.00	0.05	£ 0.00	0.05	£ 0.00	0.05	£ 0.00
SMS text message	£ 0.10	0.9	£ 0.09	0.9	£ 0.09	0.9	£ 0.09	0.9	£ 0.09	0.9	£ 0.09	0.9	£ 0.09
Treatment[7]	£ 1.80	1	£ 1.80	1	£ 1.80	1	£ 1.80	1	£ 1.80	1	£ 1.80	1	£ 1.80
Follow-up call[5]													
Blend admin/clerical[1,4]	£ 0.53	5	£ 2.64	5	£ 2.64	5	£ 2.64	5	£ 2.64	5	£ 2.64	5	£ 2.64
Nurse band 5/6[2]	£ 0.75	10	£ 7.50	10	£ 7.50	10	£ 7.50	10	£ 7.50	10	£ 7.50	10	£ 7.50
Phone call[2]	£ 0.07	3	£ 0.21	3	£ 0.21	3	£ 0.21	3	£ 0.21	3	£ 0.21	3	£ 0.21

Shaded entries were taken directly from the basic cost of a (first) chlamydia test[1] which is reproduced here under a Creative Commons licence:

© Pathway Analytics

All other costs are either amended costs from Pathway Analytics (see below for explanation), or costs added in.

[1]Any contact at a distance with client further to the initial retest conversation is assumed to require 5 minutes of admin time to retrieve and update the client's details on a database. [2]A phone call is assumed to be 3 minutes in length but requiring 10 minutes of nurse time to accommodate chasing time. [3]Applicable to clinic retesting: these costs are removed entirely where retesting involves postal testing only, and reduced

proportionally for the remaining recall methods to allow for some clients opting for postal retesting. [4]Applies to postal kits: includes postage to client's address and return postage[2], and associated admin time for sending out a kit. [5]Positives only. [6]It is assumed that all clients will received these at some point including those opting for retesting by post. [7]4 x 250mg of azithromycin in tablet form[3]. VAT, dispensing costs and costs associated with a test for cure are not included. [8]Number of units for clinic vs postal testing kit costs obtained by multiplying base costs by the percentage using each.

For peer review only

Appendix Figure 1 Retesting pathway

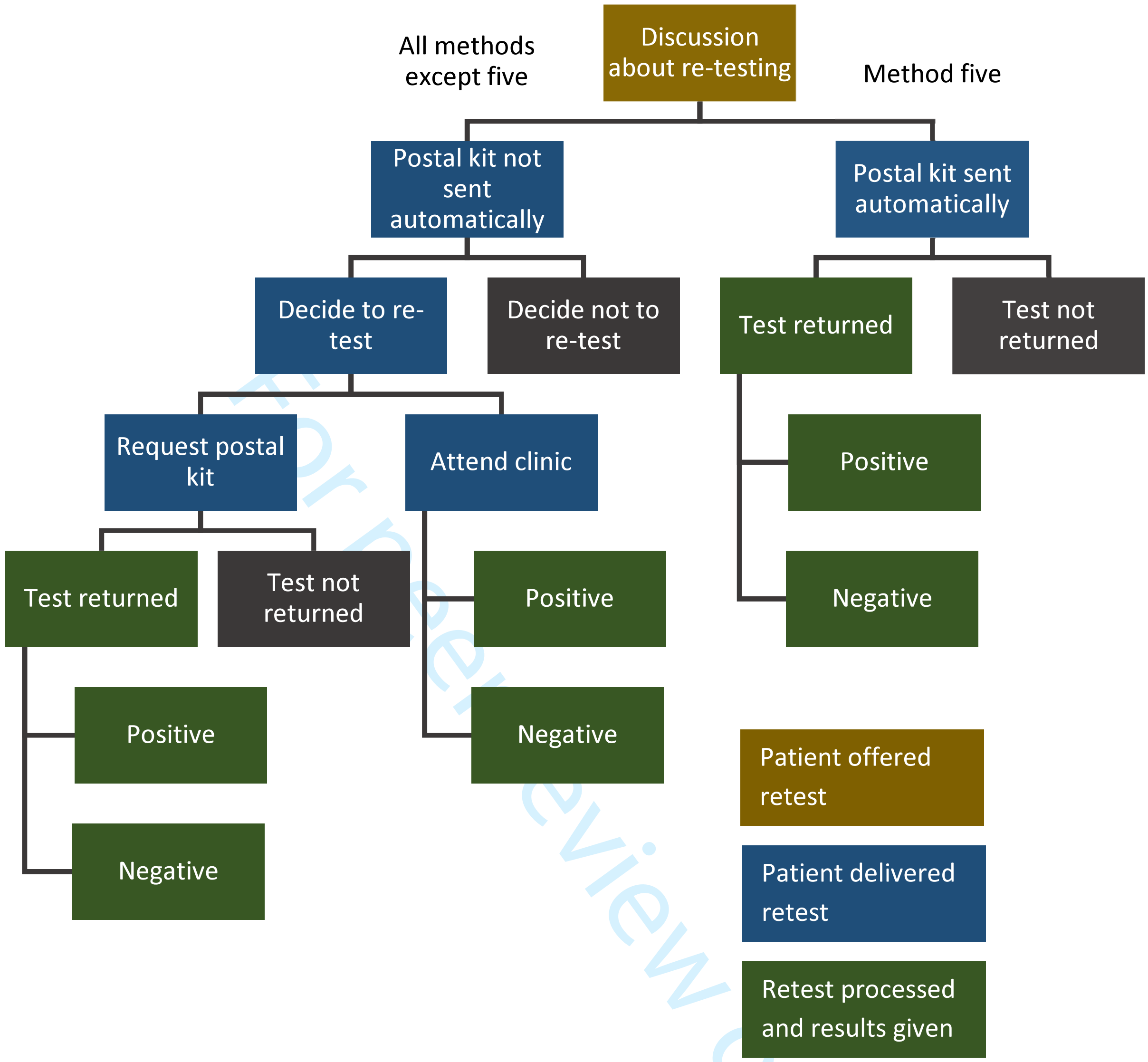

Appendix Table 2 Parameter values

Parameter	Baseline value (10-14 weeks since treatment for first infection)		Sensitivity value (10-26 weeks since treatment for first infection)		Reference
Retest uptake (i.e., percentage of (first) positives who choose to accept retesting) (all methods except method five)[1]	1. Client-led	5%	1. Client-led	15%	NCSP audit report[4] and NCSP audit data provided by PHE
	2. Reminder card	4%	2. Reminder card	19%	
	3. SMS invitation	9%	3. SMS invitation	21%	
	4. Phone invitation	7%	4. Phone invitation	17%	
	6. Advice at follow-up & SMS	13%	6. Advice at follow-up & SMS	25%	
Percentage of those retesting who choose to attend a clinic for a retest (all methods except method five)[2]	76%		73%		NCSP audit data provided by PHE
Percentage of those retesting who choose to request a postal kit (all methods except method five)	=100-76% =24%		=100-73% =27%		
Postal test kit return rate (requested kits)	67%		67%		Retesting pilot[5]
Postal test kit return rate (kits sent out automatically; method five)	5. Automatic postal test kit	10%	5. Automatic postal test kit	23%	NCSP audit report[4] and NCSP audit data provided by PHE
Chlamydia retest positivity[3]	12%		16%		NCSP audit report[4] and NCSP audit data provided by PHE

[1]Obtained by fitting to overall retest rates from the audit (i.e., accounting for non-return of requested postal kits); [2]The audit only has data on percentage of *completed* retests obtained from clinic testing vs postal testing (for those instances where a kit was not sent out automatically), not percentage of those who opt for a retest at a clinic among *all* retesters (i.e., including all those who request a kit, some of whom do not return the kit); [3]Average over the six most commonly-used methods.

References

1. Pathway Analytics: Pathway Prices for Integrated Sexual Health Tariffs *118. T3 Chlamydia, gonorrhoea and syphilis tests* https://www.pathwayanalytics.com/pathways/26-t3-chlamydia-gonorrhoea-syphilis-tests/118-t3-chlamydia-gonorrhoea-and-syphilis-tests *Accessed 2013.*
2. *Royal Mail: Get a price* http://www.royalmail.com/price-finder *Accessed 14/08/2015.*
3. *British National Formulary: Azithromycin* http://www.evidence.nhs.uk/formulary/bnf/current/5-infections/51-antibacterial-drugs/515-macrolides/azithromycin *Accessed 02/02/2016.*
4. *Re-testing of those who tested positive for chlamydia: National audit report* https://www.gov.uk/government/uploads/system/uploads/attachment_data/file/471585/NCSPre-testingauditfinalversion.pdf *Accessed 13/09/2016.*
5. Angel, G., et al., *An observational study to evaluate three pilot programmes of retesting chlamydia-positive individuals within 6 months in the South West of England.* BMJ Open, 2016. **5**(10): p. e007455.

For peer review only

CHEERS checklist—Items to include when reporting economic evaluations of health interventions (BMJ 2013;346:f1049)

Section/item	Item No	Recommendation	Reported on page No/ line No
**Title and abstract**			
Title	1	Identify the study as an economic evaluation or use more specific terms such as “cost-effectiveness analysis”, and describe the interventions compared.	p1/line 1-3
Abstract	2	Provide a structured summary of objectives, perspective, setting, methods (including study design and inputs), results (including base case and uncertainty analyses), and conclusions.	p2
**Introduction**			
Background and objectives	3	Provide an explicit statement of the broader context for the study.	p3/line 67-73
		Present the study question and its relevance for health policy or practice decisions.	p3-4/line 89-96
**Methods**			
Target population and subgroups	4	Describe characteristics of the base case population and subgroups analysed, including why they were chosen.	N/A
Setting and location	5	State relevant aspects of the system(s) in which the decision(s) need(s) to be made.	p4/line 98
Study perspective	6	Describe the perspective of the study and relate this to the costs being evaluated.	p4/line 98-100
Comparators	7	Describe the interventions or strategies being compared and state why they were chosen.	p4/line 98-100

Section/item	Item No	Recommendation	Reported on page No/ line No
Time horizon	8	State the time horizon(s) over which costs and consequences are being evaluated and say why appropriate.	p4/line 98-102
Discount rate	9	Report the choice of discount rate(s) used for costs and outcomes and say why appropriate.	N/A
Choice of health outcomes	10	Describe what outcomes were used as the measure(s) of benefit in the evaluation and their relevance for the type of analysis performed.	N/A
Measurement of effectiveness	11a	*Single study-based estimates:* Describe fully the design features of the single effectiveness study and why the single study was a sufficient source of clinical effectiveness data.	N/A
	11b	*Synthesis-based estimates:* Describe fully the methods used for identification of included studies and synthesis of clinical effectiveness data.	N/A
Measurement and valuation of preference based outcomes	12	If applicable, describe the population and methods used to elicit preferences for outcomes.	N/A
Estimating resources and costs	13a	*Single study-based economic evaluation:* Describe approaches used to estimate resource use associated with the alternative interventions. Describe primary or secondary research methods for valuing each resource item in terms of its unit cost. Describe any adjustments made to approximate to opportunity costs.	p4/line 100-110 and Appendix Table 1
	13b	*Model-based economic evaluation:* Describe approaches and data sources used to estimate	N/A

Section/item	Item No	Recommendation	Reported on page No/ line No
		resource use associated with model health states. Describe primary or secondary research methods for valuing each resource item in terms of its unit cost. Describe any adjustments made to approximate to opportunity costs.	
Currency, price date, and conversion	14	Report the dates of the estimated resource quantities and unit costs. Describe methods for adjusting estimated unit costs to the year of reported costs if necessary. Describe methods for converting costs into a common currency base and the exchange rate.	pP4/line 100-102
Choice of model	15	Describe and give reasons for the specific type of decision-analytical model used. Providing a figure to show model structure is strongly recommended.	Appendix Figure 1
Assumptions	16	Describe all structural or other assumptions underpinning the decision-analytical model.	p4/line 104-121 and Appendix Table 2
Analytical methods	17	Describe all analytical methods supporting the evaluation. This could include methods for dealing with skewed, missing, or censored data; extrapolation methods; methods for pooling data; approaches to validate or make adjustments (such as half cycle corrections) to a model; and methods for handling population heterogeneity and uncertainty.	N/A
**Results**			
Study parameters	18	Report the values, ranges, references, and, if used, probability distributions for all parameters. Report reasons or sources for distributions used to represent	p4/line 104-121 and Appendix Table 2

Section/item	Item No	Recommendation	Reported on page No/ line No
		uncertainty where appropriate. Providing a table to show the input values is strongly recommended.	
Incremental costs and outcomes	19	For each intervention, report mean values for the main categories of estimated costs and outcomes of interest, as well as mean differences between the comparator groups. If applicable, report incremental cost-effectiveness ratios.	Table 1
Characterising uncertainty	20a	*Single study-based economic evaluation:* Describe the effects of sampling uncertainty for the estimated incremental cost and incremental effectiveness parameters, together with the impact of methodological assumptions (such as discount rate, study perspective).	N/A
	20b	*Model-based economic evaluation:* Describe the effects on the results of uncertainty for all input parameters, and uncertainty related to the structure of the model and assumptions.	Table 1
Characterising heterogeneity	21	If applicable, report differences in costs, outcomes, or cost-effectiveness that can be explained by variations between subgroups of patients with different baseline characteristics or other observed variability in effects that are not reducible by more information.	N/A
**Discussion**			
Study findings, limitations, generalisability, and current knowledge	22	Summarise key study findings and describe how they support the conclusions reached. Discuss limitations	p7-8

Section/item	Item No	Recommendation	Reported on page No/ line No
		and the generalisability of the findings and how the findings fit with current knowledge.	
**Other**			
Source of funding	23	Describe how the study was funded and the role of the funder in the identification, design, conduct, and reporting of the analysis. Describe other non-monetary sources of support.	p9
Conflicts of interest	24	Describe any potential for conflict of interest of study contributors in accordance with journal policy. In the absence of a journal policy, we recommend authors comply with International Committee of Medical Journal Editors recommendations.	P8

For consistency, the CHEERS statement checklist format is based on the format of the CONSORT statement checklist

For peer review only - http://bmjopen.bmj.com/site/about/guidelines.xhtml
